# A Comparative Study on Asymmetric Reduction of Ketones Using the Growing and Resting Cells of Marine-Derived Fungi

**DOI:** 10.3390/md16020062

**Published:** 2018-02-14

**Authors:** Hui Liu, Bi-Shuang Chen, Fayene Zeferino Ribeiro de Souza, Lan Liu

**Affiliations:** 1 School of Marine Sciences, Sun Yat-Sen University, Guangzhou 510275, China; liuh229@mail.sysu.edu.cn (H.L.); cesllan@mail.sysu.edu.cn (L.L.); 2 Guangdong Provincial Key Laboratory of Marine Resources and Coastal Engineering, Guangzhou 510275, China; 3 South China Sea Bio-Resource Exploitation and Utilization Collaborative Innovation Center, Sun Yat-Sen University, Guangzhou 510275, China; 4 Departamento de Química, Faculdade de Ciências, UNESP, Bauru 17033-360, Brazil; faylittlefay@yahoo.com.br

**Keywords:** growing cells, resting cells, asymmetric reduction, marine fungi, chiral alcohols

## Abstract

Whole-cell biocatalysts offer a highly enantioselective, minimally polluting route to optically active alcohols. Currently, most of the whole-cell catalytic performance involves resting cells rather than growing cell biotransformation, which is one-step process that benefits from the simultaneous growth and biotransformation, eliminating the need for catalysts preparation. In this paper, asymmetric reduction of 14 aromatic ketones to the corresponding enantiomerically pure alcohols was successfully conducted using the growing and resting cells of marine-derived fungi under optimized conditions. Good yields and excellent enantioselectivities were achieved with both methods. Although substrate inhibition might be a limiting factor for growing cell biotransformation, the selected strain can still completely convert 10-mM substrates into the desired products. The resting cell biotransformation showed a capacity to be recycled nine times without a significant decrease in the activity. This is the first study to perform asymmetric reduction of ketones by one-step growing cell biotransformation.

## 1. Introduction

The preparation of enantiomerically pure secondary alcohols is of ever-increasing significance because these intermediates are important building blocks for the production of chiral pharmaceuticals, flavours, agrochemicals and functional materials [1,2]. For example, (*S*)-1-(3,4-dichlorophenyl)ethan-1-ol (**2n**) is a versatile intermediate in the synthesis of sertraline [3,4], which is used to treat depression, obsessive-compulsive disorder, panic disorder, anxiety disorders, post-traumatic stress disorder, and premenstrual dysphoric disorder. Another optically active alcohol, ethyl (*S*)-4-chloro-3-hydroxybutanoate, is a key intermediate for the synthesis of 3-hydroxy-3-methylglutaryl coenzyme A (HMG-CoA) reductase inhibitors, which are the active ingredients of a cholesterol-lowering drug Lipitor [5], while 2-bromo-1-phenylethanol (**2a**) is the precursor for the synthesis of anti-depressants and α- or β-adrenergic drugs, such as fluoxetine, tomoxetine, and nisoxetine [6,7].

While there are many synthetic approaches that furnish chiral alcohols in high enantiomeric excess, catalytic asymmetric reduction of prochiral carbonyl compounds offers several advantages because it can easily be applied to a wide range of substrates, the utilization of material is high, and waste and by-products can be drastically reduced [8,9]. Chemical methods using metal-complex catalysts have been employed [10,11,12]. Nevertheless, due to the presence of metals, most of the described methods require the use of either a cumbersome catalyst preparation or reductive/oxidative follow-up chemistry. Metal-free catalytic enantioselective reduction of prochiral ketones remains challenging.

Biocatalysis in asymmetric synthesis, as a result of their complex chiral constitution, are predominantly suited for the manufacture of optically pure stereoisomers [13,14,15,16,17]. Indeed, bioreduction of ketones using carbonyl reductases to chiral alcohols have been found and purified, such as S1 from *Candida magnoliae* [18], KaCR from *Kluyveromyces aestuarii* [19], and PsCR I from *Pichia stipitis* [20]. However, the high price of cofactors (approximately 1 g/485 euros), including Nicotinamide adenine dinucleotide (NADH) or Nicotinamide adenine dinucleotide phosphate (NADPH), is an impediment to the application of this approach. Therefore, efficient and cost-effective cofactor regeneration systems such as enzyme- and substrate-coupled systems must be developed [21]. Formate dehydrogenase (FDH) or glucose dehydrogenase (GDH) could be used as enzyme-coupled systems for the recycling of NAD^+^ or NADP^+^ [22], and 2-propanol can be used as a co-substrate because of its low cost and the feasibility of forcing the reaction towards completion by removing the acetone co-product under reduced pressure [23,24].

An easily operated and whole-cell system adaptable method is regarded as a viable “green” alternative synthetic approach [25,26] due to its unique advantages such as mild reaction conditions, environmental friendliness, regeneration of cofactors in situ, easy production and relatively low price; this method has therefore attracted great attention and been extensively investigated in recent years [27]. However, most whole-cell catalytic studies involve resting cells rather than growing cell biotransformation [28,29,30]. The resting cells are resuspended in buffer solution under non-growing conditions and are used as biocatalysts for the production of target compounds, which benefit from convenient downstream product separation. Growing cell biotransformation is the one-step process in which a certain amount of substrate was added to the medium and the target product was synthesized via one or several enzymatic reactions from the substrate during cell culture. Growing cell biotransformation is similar to microbial fermentation in its potential for industrial-scale production and shows significant advantages over resting cell biotransformation due to its ease of execution, which is a result of features such as simple operation steps, no need for cell preparation, and readiness for industrial scale production.

Therefore, in the present study, we report the results of a comparative study on the asymmetric reduction of a variety of aromatic ketones using growing and resting cells of marine-derived fungi that offers an alternative, highly enantioselective and minimally polluting route to important enantiomeric pure alcohols.

## 2. Results

To fully assess the potential of marine-derived fungi as biocatalysts for the enantioselective reduction of prochiral ketones, whole mycelia of 13 marine fungi (*Penicillium citrinum* GIM 3.458, *Penicillium citrinum* GIM 3.251, *Penicillium citrinum* GIM 3.100, *Aspergillus sclerotiorum* AS 3.2578, *Aspergillus sydowii* AS 3.7839, *Aspergillus sydowii* AS 3.6412, *Geotrichum candidum* GIM 2.361, *Geotrichum candidum* GIM 2.616, *Rhodotorula rubra* GIM 2.31, *Rhodotorula mucilaginosa* GIM 2.157, *Geotrichum candidum* AS 2.1183, *Geotrichum candidum* AS 2.498 and *Rhodotorula rubra* AS 2.2241) were screened for stereoselective reduction of 1-(3-bromophenyl)ethan-1-one **1b**. The screening reaction was initially performed with 10 mL of Na_2_HPO_4_-KH_2_PO_4_ buffer (100 mM, pH 6.0) containing glucose (0.5 g), 5 mM 1-(3-bromophenyl)ethan-1-one (**1b**), and 3 g of resting cells at 30 °C, due to its frequent use for described resting cell biotransformation [31,32,33,34]. The results are shown in Table 1. The absolute configuration of (*S*)-**2b** was determined by comparing the specific measured signs of rotation to those reported in the literature [35]. In a control reaction performed in parallel and involving only (**1b**), glucose and buffer (without resting cells), no yield of the desired product (*S*)-1-phenylpropan-1-ol [(*S*)-**2b**] was detected, indicating that no chemically catalysed reaction occurred; thus, the reaction was carried out by the active enzymes present in the marine-derived fungi. It must be emphasized that the complete genome of strain *Rhodotorula rubra* AS 2.2241 has been sequenced and annotated in our laboratory. Taking the conversion, enantioselectivity, and availability of the genome sequence into account, we decided to continue to use strain *R. rubra* AS 2.2241 for all further studies.

### 2.1. Reductions with Growing Cells

#### 2.1.1. Optimization of Growing Cell Biotransformation

Substrate **1b** was added at the time of inoculation. From Table 2, it is evident that *R. rubra* AS 2.2241 could grow in the presence of substrate **1b** and could reduce substrate **1b** into the corresponding alcohol (*S*)-**2b**. The growth of *R. rubra* AS 2.2241 in the presence of substrate **1b** (entries 1–5) was less than in the control media without ketone (entry 2′). Thirty-five percent less growth was observed in the medium with 5 mM substrate **1b**. With 10 mM, 11.6 mM and 13.3 mM ketone in the cultivation medium, 60%, 84% and 93% less growth was observed, respectively. There was no growth of *R. rubra* AS 2.2241 with 15 mM ketone **1b** in the medium. It appears that ketone **1b** had an inhibitory effect on the growth of *R. rubra* AS 2.2241, and the inhibition increases with the increasing concentration of ketone **1b**. No transformation of ketone **1b** was observed in the flask that was not inoculated with *R. rubra* AS 2.2241 (control 1′). This indicated that ketone **1b** could not be reduced into alcohols without the organism. The second control contained only the RM1 medium without ketone **1b**, and the flask was inoculated (control 2′). No corresponding alcohol **2b** was observed, suggesting that alcohol **2b** was a metabolism-independent product, and this reaction was an enzyme-catalytic reduction. Heat-denatured cell preparations in control experiments (control 3′) clearly showed that no chemically catalysed reaction occurred, thus providing further support for the fact that the reaction is carried out by the active enzyme. Although the growth of *R. rubra* AS 2.2241 in the presence of ketone **1b** was lower, it could grow in the presence of ketone **1b** and reduce ketones **1b** into alcohols (*S*)-**2b**.

The next experiment was set up to determine the minimum growth of *R. rubra* AS 2.2241 required for the transformation of ketone **1b**. Here, different concentrations of ketones (5–15 mM) were added to the RM1 medium and inoculated with *R. rubra* AS 2.2241. The flasks were shaken (220 rpm) at 28 °C, and the samples were analysed at 24-h intervals. It was found that when the substrate concentration was smaller than 13.3 mM, the bioreduction reaction rate showed a clear trend towards an increase in product formation over time during the first 72 h of the reaction. The reaction velocity as well as product formation was low at the initial stage owing to low carbonyl reductase expression and then increased at the later stage, even though the desired product formation decreased over 72 h to 96 h. This decrease may be due to the alcohol dehydration, as was also found by Chandran and Das [36]. No significant changes in the product *ee* (almost above 99%) within the study were observed. When the substrate concentration was above 13.3 mM, cell growth and production of desired alcohols were not observed, suggesting the existence of substrate inhibition during the growing cell transformation. Therefore, the optimal substrate concentration in the medium was 10 mM. To date, yeasts, bacteria, fungi, and even plant tissues have been employed as biocatalysts for bio-reduction processes [28,29,30]. However, most of these biotransformations were performed with the resting cells of these microorganisms. Direct addition of substrates to the medium (one-pot) has significant potential for industrial scale production of chemicals because of the simplified cell preparation. Moreover, the total time of incubation was relatively short because growth and biotransformation occurred simultaneously. Although the substrates showed an inhibitory effect on the growth of *R. rubra* AS 2.2241, this growing cell biotransformation still showed good productivity in asymmetric reduction of tested ketones.

**Figure 1:**
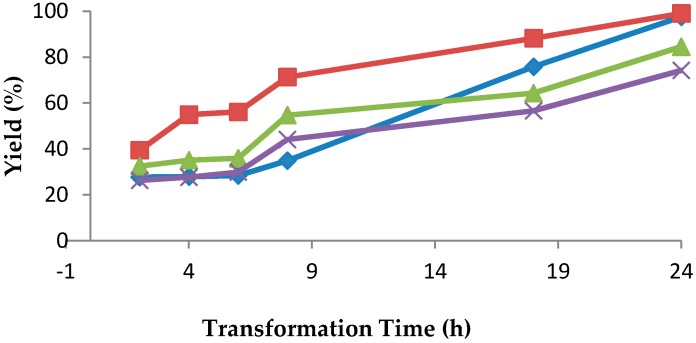
Effects of age of growing cells of *R. rubra* AS 2.2241 on the transformation of ketone **1b** (transformation was carried out with the growing cells of *R. rubra* AS 2.2241, and ketone **1b** was added at the different phases of growth: blue represents the data for **1b** added at 24 h of growth; red represents the data for **1b** added at 48 h of growth; green represents the data for **1b** added at 72 h of growth; purple represents the data for **1b** added at 96 h of growth).

Substrate 1b was added at different phases of growth. Addition of substrates at the beginning of the fermentation caused growth inhibition; therefore, substrate feeding (10 mM) was performed after induction. As illustrated in Figure 1, the 24-h-grown growing cells of *R. rubra* AS 2.2241 reduced ketone **1b** into alcohol (*S*)-**2b** within 24 h of incubation, while 48-h-grown growing cells also took 24 h to complete the transformation. In the case of 72-h-grown growing cells, the transformation was slightly smaller, and 84% of the added ketone **1b** was transformed after 24 h of incubation. Only 74% of ketone **1b** was transformed by 96-h-grown growing cells after 24 h of incubation. No further transformation was noticed with 96-h-grown cells with a prolonged period of incubation. In all cases, the initial ketones **1b** concentration was 10 mM. Thus, it is clear from these experiments that 24- and 48-h-grown growing cells were most active in transforming ketone **1b** to alcohol (*S*)-**2b**. This may be due to the presence of active enzymes after 24 and 48 h of growth. It has also been reported that maximum carbonyl reductase activity was obtained within 45–50 h of cultivation of *Acetobacter pasteurianus* GIM1.158 [37]. Poor enzyme activity was observed in 72-h-grown and 96-h-grown cultures, making the transformation of ketone **1b** inefficient. Poor enzyme activity may be due to the production of some metabolites that inactivated the enzyme. Transformation of ketone **1b** with growing cells used much more enzyme than that with the soluble enzyme system, but the time required for the transformation with the growing cells was not shorter. This is possible because the enzymatic system with the growing cells worked in an uncleaned environment (with cells, unused substrates and metabolites, etc.).

#### 2.1.2. Growing Cell Biotransformation of Ketones **1a**–**1n**

**Figure 2:**
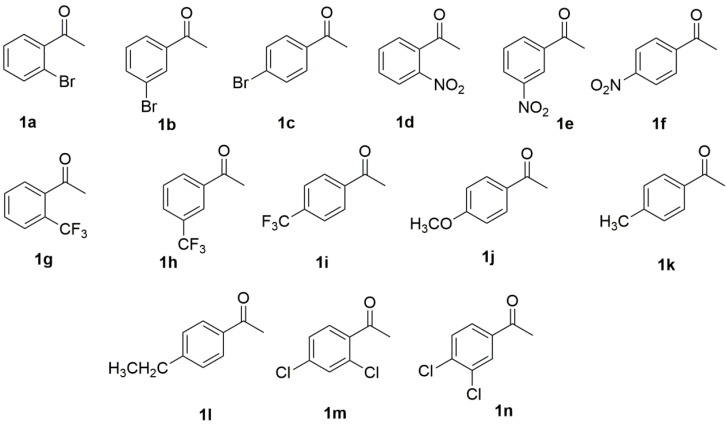
Structurally related aromatic ketones used for bioreduction by growing cells of *R. rubra* AS 2.2241.

We next turned our attention to the substrates scope and the limitation of growing cells of strain *R. rubra* AS 2.2241 for the reduction of prochiral ketones **1a**–**1n**. Another 13 aromatic ketones that were structurally closely related to the main test substrate 1b were tested (Figure 2) under the optimized conditions (addition of 10 mM substrates at the beginning of the fermentation and incubation for 72 h at 28 °C in RM1 medium buffer at pH 7.0), with the results presented in Table 3. Since the corresponding racemic reduction products (±)-β-phenylalcohols **2a**–**2m** (except **2b**) were not commercially available, they were obtained by the reduction of the aromatic ketones **1a**, **1c**–**1m** with sodium borohydride in methanol [38] and used as standard compounds for the analysis of the bioreduction products via chiral HPLC. The NMR spectra are in agreement with those reported in the literature [35,39,40,41,42,43].

For substrates (**1a**–**1i**) with an electron-withdrawing group such as –Br, –NO_2_, –CF_3_ in either *ortho*-, or *meta*-, or *para*-position (Table 3, entries 1–9), the reaction proceeded smoothly in all cases to yield the corresponding reduction products with more than 99% yield and 99% *ee*. By contrast, none of substrates (**1j**, **1k**, **1l**) with an electron-releasing group in the para-position were accepted by the enzyme (Table 3, entries 10–12), suggesting that an electron-withdrawing group might play an important role for the proper orientation of the ketones in the enzymes’ active site. However, when substrates with electron-withdrawing groups in both ortho- and para-positions (**1m**) or in both meta- and para-positions (**1n**) were used, no desired reduction products were obtained, which is probably due to their more bulky structures. The absolute configurations of the reduction products (**2a**–**2i**) were determined by comparing the specific measured signs of rotation to those reported in the literature [35,39,40,41,42,43].

### 2.2. Reductions with Resting Cells

#### 2.2.1. Optimization of Resting Cell Biotransformation

The transformation of ketone **1b** using the resting cells of different ages showed different types of transformation patterns. Here, the cells were grown under growth conditions in a nutrient medium for 24, 48, 72 and 96 h, and the cells were harvested from each phase and washed several times with a phosphate (0.1 M, pH 7.0) buffer. The strict separation of microbial growth and biotransformation offers many advantages: the washed cells were suspended to buffer (100 mM, pH 7.0) to transform ketone **1b** (10 mM) at 28 °C in an orbital shaker (220 rpm). The effects of the temperatures (in the range of 20–35 °C) and pH values (pH 6–8) on biotransformation were evaluated using the resting cells collected after 48 h of growth. As shown in Table 4, 24-h-grown resting cells gave the highest conversion (89% yield, 99% *ee*) at 25 °C with pH 7.0 buffered by Na_2_HPO_4_-KH_2_PO_4_ (100 mM) after 24 h. Therefore, these reaction conditions were used to further test the activity of the resting cells grown for 48 h, 72 h and 96 h. Gratifyingly, in each case, ketone **1b** was reduced into (*S*)-**2b** with good yields and excellent enantioselectivities. The 48-h-grown resting cells showed the slightly better activity than those obtained for the 72-h-grown and 96-h-grown resting cells, and the 48-h-grown resting cells were chosen for all further studies of the resting cell biotransformation.

**Figure 3:**
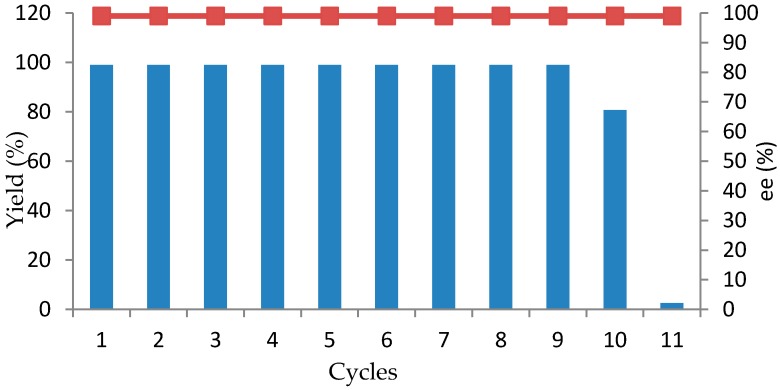
Reusability of resting cells of *R. rubra* AS 2.2241 of 48 h-grown age in the transformation of ketone **1b** (in each case the initial concentration of **1b** was 10 mM). Bar represents yields, square represents *ee* values. Yield and *ee* values were determined by chiral HPLC.

One of the important considerations for industrial production is the capacity to recycle the catalyst. Therefore, experiments were performed to examine the recyclability of the resting cells of *R. rubra* AS 2.2241 for the reduction of **1b** as an example. For the results summarized in Figure 3, every reaction was performed in 10 mL of Na_2_HPO_4_-KH_2_PO4 buffer (100 mM, pH 7.0) with 3 g of wet cells, 10 mM substrate and 0.5 g of glucose and shaken at 25 °C for 23 h. At the end of the reaction, the cells were centrifuged, washed twice with the same buffer [Na_2_HPO_4_-KH_2_PO_4_ buffer (100 mM, pH 7.0)] and reused for the next cycle under the same reaction conditions. Resting cells of strain *R. rubra* AS 2.2241 exhibited high activity and complete conversion for nine cycles (Figure 3). Only a slight decrease was observed in cycle 10, whereas almost no activity (2.6% yield of the desired product) was found in cycle 11. Remarkably, the reduction product *ee* showed no significant variation and remained above 99%. The high reusability of the whole cells of the strain *R. rubra* AS 2.2241 could be attributed to the expression of naturally immobilized ketone reductase in the cells, which is currently being implemented in our laboratory.

#### 2.2.2. Resting Cell Biotransformation of Ketones **1a**–**1n**

Under the condition optimized for the resting cell biotransformation, 14 substrates shown in Figure 2 were again used to test the substrate scope and limitations. As shown in Table 5, for most substrates (**1a**–**1l**, Table 5, entries, 1–12), the same results were obtained as those obtained with growing cell biotransformation. Surprisingly, substrates **1m** and **1n** were also converted by resting cells, and the corresponding products were obtained in 63% yield with 28% *ee* and 55% yield with 99% *ee*, suggesting that there might be different enzymes present in the resting cells which could be able to accept the substrates with bulky structure. The production of some metabolites during growing cell biotransformation inactivated the enzyme; therefore, there was no conversion for substrates **1m** and **1n**, as found by Banerjee [44] for the comparison of the activity of oxidases of *Curvularia lunata* in growing cell biotransformation and resting cell biotransformation.

## 3. Discussion

The development of biocatalysis requires novel biocatalysts in the form of isolated enzymes or whole cells [45], leading to a growing demand for robust and efficient biocatalysts. Fungi from marine environments are thoroughly adapted to surviving and growing under harsh conditions [46]. Such habitat-related characteristics are desirable features from a general biotechnological perspective and are of key importance to exploit a microorganism’s enzymatic potential [47,48,49,50]. Indeed, fungi host novel enzymes showing optimal activities at extreme values of salt concentrations, pH and temperature, compared to enzymes isolated from terrestrial origins [51,52,53]. These advantages, in addition to their chemical and stereochemical properties and readily available sources (e.g., sea sources of enzymes represented by microorganisms or fungi, plants or animals; ease of growth), make marine enzymes ideal biocatalysts for fine chemistry and pharmaceutical sectors; these enzymes should be broadly explored [28,29,30,48,54,55,56].

Growing cells are under the growing conditions of proliferating and metabolically active cells, which are suspended in the medium of essential nutrients. Growing cell biotransformation is similar to the microbial fermentation, in which a certain amount of substrate was added at the time of inoculation or at regular phases of growth and was converted into the desired product via one or several enzymatic reactions during cell culture. The biotransformation is stable due to suitable growing environment for the cells. This one-step biotransformation showed substantial advantages such as the simultaneous growth and biotransformation, and simple operation steps, but usually substrate had an inhibition effect on the growing of cells which might be a limiting factor for growing cell biotransformation. Therefore, a straightforward approach for bioreduction of carbonyl compounds into the desired enantiomerically chiral alcohols using growing cells biotransformation still had not been described.

The resting cells refer to the group of active cells which are provided in buffer solution under non-growing conditions without any nutrients and are solely used as biocatalysts to yield many compounds [57]. Resting cell biotransformation show certain advantages over growing cell biotransformation, including no limit of substrate concentrations and convenient down downstream product separation. Besides, modelling and simulation of resting cell biotransformation processes had been successfully used to understand the investigated process, identify the limiting parameters, and optimize the reaction conditions. However, for industrial application, growing cells biotransformation showed more efficiency than that of resting cells biotransformation due to cumbersome catalyst preparation and storage.

Thus, we compared the productivity of marine fungi *R. rubra* AS 2.2241 from one-step growing cell biotransformation and two-step resting cell biotransformation in enantiomeric pure alcohols. Both of the reaction systems were carefully optimized for 10 mM-scale synthesis, resulting in good conversions and excellent enantioselectivities. Under the optimized conditions, *R. rubra* AS 2.2241 could grow in the presence of ketones **1a**–**1i** and could convert them into the corresponding alcohols (*S*)-**2a**–**2i**, offering an attractive alternative to the synthesis of enantiopure alcohols. Resting cells of different ages were also very effective in transforming ketones **1a**–**1i**, including **1m** and **1n**. The 48-h-grown resting cells could be reused for nine cycles without significant loss of activity while maintaining up to 99% *ee*. Further work will be focused on the elimination of substrate inhibition to improve the production and cell growth in growing cell biotransformation.

## 4. Materials and Methods

### 4.1. General

All chemicals were purchased from Sigma–Aldrich (Schnelldorf, Germany) and used without further purification unless specified otherwise. The culture media components were obtained from BD (Becton, Dickinson and Company, Bremen, Germany).

^1^H and ^13^C NMR spectra were recorded using a Bruker Advance 400 (Karlsruhe, Germany) (400 MHz and 100 MHz, respectively) instrument and internally referenced to residual solvent signals. Data for ^1^H NMR are reported as chemical shift (d ppm), multiplicity (s = singlet, d = doublet, t = triplet, q = quartet, m = multiplet), integration, coupling constant (Hz) and assignment. Data for ^13^C NMR are reported in terms of chemical shift. Optical rotations were obtained at 20 °C using a PerkinElmer 241 polarimeter (Shanghai, China) (sodium D line). Column chromatography was performed with a silica gel (0.060–0.200 mm, pore diameter ca. 6 nm) and mixtures of petroleum ether (PE) and ethyl acetate (EtOAc) as solvents. Thin-layer chromatography (TLC) was performed on 0.20 mm silica gel 60-F plates. Organic solutions were concentrated under reduced pressure with a rotary evaporator.

### 4.2. Chemical Synthesis of the Standard Racemic β-Phenylalcohols ***2a***–***2f***, ***2m*** and ***2n***

Ten mmol of NaBH_4_ was added to a cooled (0 °C) solution of 2.5 mmol of each specific substrate (**1a**–**1f**, **1m** and **1n**) in 50 mL of methanol. After stirring for 10 min, the mixture was warmed to room temperature and stirred for another 3–4 h to complete the reduction. After quenching with 2 M HCl to pH 7.0, the mixture was extracted with EtOAc (50 mL × 3). The organic phases were washed with brine, dried over Na_2_SO_4_, filtered and concentrated in vacuum. The residue was purified by flash chromatography on silica gel (eluent: EtOAc/PE 1:20) to give the racemic alcohol **2a**–**2f**, **2m** and **2n** (see Supplementary Materials for NMR spectroscopic data).

### 4.3. Microorganisms

The marine fungi strains *Penicillium citrinum* GIM 3.458, *Penicillium citrinum* GIM 3.251, *Penicillium citrinum* GIM 3.100, *Aspergillus sclerotiorum* AS 3.2578, *Aspergillus sydowii* AS 3.7839, *Aspergillus sydowii* AS 3.6412, *Geotrichum candidum* GIM 2.361, *Geotrichum candidum* GIM 2.616, *Rhodotorula rubra* GIM 2.31, *Rhodotorula mucilaginosa* GIM 2.157, *Geotrichum candidum* AS 2.1183, *Geotrichum candidum* AS 2.498 and *Rhodotorula rubra* AS 2.2241 were isolated from a wide collection of isolates from marine sediments from Guangdong Province, China. All strains used in this study were deposited and commercially available at Guangdong Culture Collection Center or the China General Microbiological Culture Collection Center.

The marine fungi were maintained on agar plates at 4 °C and subcultured at regular intervals. The medium (RM1) used for cultivation contained glucose (15 g/L), peptone (5 g/L), yeast extract (grease, 5 g/L), disodium hydrogen phosphate (0.5 g/L), sodium dihydrogen phosphate (0.5 g/L), magnesium sulphate (0.5 g/L) and sodium chloride (10 g/L) and a final pH 7.0; this medium was sterilized at 115 °C in an autoclave for 25 min. A loop of a single colony was cut from the agar stock cultures and used to inoculate a given medium in an appropriate Erlenmeyer flask. This culture was shaken reciprocally (220 ppm) at 28 °C for given times.

### 4.4. Transformation with Growing Cells

Growing cells of *R. rubra* AS 2.2241 were used for the transformation of 1-(3-bromophenyl)ethanone (**1b**). 1-(3-bromophenyl)ethanone (**1b**) was added at the time of inoculation of the RM1 medium as described above by *R. rubra* AS 2.2241; in another set of experiments, 1-(3-bromophenyl)ethanone (**1b**) was added to the growing cells at different phases of growth, and the transformation continued.

#### 4.4.1. **1b** Was Added at the Time of Inoculation

Different concentrations (5–15 mM) of 1-(3-bromophenyl)ethanone (**1b**) were added aseptically to 500-mL Erlenmeyer flasks containing 250 mL of sterile RM1 medium (pH 7.0). Three control flasks were prepared. The first control was prepared with 10 mM 1-(3-bromophenyl)ethanone in 250 mL of RM1 medium, and the flask was not inoculated with *R. rubra* AS 2.2241. This was used as a positive control for 1-(3-bromophenyl)ethanone (**1b**). The second control contained only the RM1 medium without 1-(3-bromophenyl)ethanone (**1b**), and the flask was inoculated with *R. rubra* AS 2.2241. This was done to check the growth of *R. rubra* AS 2.2241 in the absence of 1-(3-bromophenyl)ethanone (**1b**). The third control was prepared with 5 g of (dry weight) dead cells of *R. rubra* AS 2.2241 in RM1 medium with 10 mM 1-(3-bromophenyl)ethanone (**1b**). This was used to determine the active enzymes by the cells. This flask was not inoculated. All flasks were kept in a temperature-controlled (28 °C) orbital shaker at 220 rpm shaking speed. The samples were analysed after 24 h. The cells were removed by centrifugation at 4000 rpm and at 4 °C for 20 min. The collected cells were used for cell mass analysis. The supernatant (2 mL) was saturated with NaCl followed by extraction with 2 × 2 mL of HPLC eluent (*n*-hexane/*i*-PrOH = 95/5, *v/v*) by shaking for 5 min. The combined organic layer was dried over Na_2_SO_4_ and measured by HPLC for yield and *ee*.

#### 4.4.2. **1b** Were Added at Different Phases of Growth

Several 500-mL Erlenmeyer flasks containing 250 mL of RM1 medium (pH 7.0) were inoculate with *R. rubra* AS 2.2241 and cultured at 28 °C on an orbital shaker (220 rpm). Next, 10 mM 1-(3-bromophenyl)ethanone (**1b**) was added to the growing cells after 24, 48, 72, 96 h of inoculation in separated flasks. For the transformation reaction, the flasks were again incubated under the same conditions. The samples were withdrawn at regular intervals and centrifuged, and the supernatant (2 mL) was saturated with NaCl followed by extraction with 2 × 2 mL of HPLC eluent (*n*-hexane/*i*-PrOH = 95/5, *v/v*) by shaking for 5 min. The combined organic layer was dried over Na_2_SO_4_ and measured by HPLC for yield and *ee*.

### 4.5. General Methods for Growing Cells Biotransformation

All substrates **1a**–**1n** were added at the time of inoculation, and the reaction setup was the same as “**1b** was added at the time of inoculation”. After a 72-h inoculation, the reaction was stopped by centrifugation at 4000 rpm and at 4 °C for 20 min to remove the cells. The supernatant (2 mL) was saturated with NaCl followed by extraction with 2 × 2 mL of HPLC eluent (*n*-hexane/*i*-PrOH = 95/5, *v/v*) by shaking for 5 min. The combined organic layer was dried over Na_2_SO_4_ and measured by HPLC for yield and *ee*.

### 4.6. Transformation with Resting Cells

*R. rubra* AS 2.2241 was cultivated in RM1 medium (pH 7.0) at 28 °C with 220 rpm speed. Cells in the culture age of 24 h, 48 h, 72 h and 96 h were harvested by centrifugation and washed twice with 100 mM Na_2_HPO_4_-KH_2_PO_4_ buffer (pH 7.0). Approximately 3 g of resting cells of 24-h-grown age was suspended in 10 mL of Na_2_HPO_4_-KH_2_PO_4_ buffer with the required pH (pH 6.0, 7.0 and 8.0) containing 0.5 g of glucose and 10 mM 1-(3-bromophenyl)ethanone (**1b**). The reaction mixtures were shaken (220 rpm) at the given temperatures (20 °C, 25 °C and 35 °C) for 24 h and stopped by centrifugation at 4000 rpm and at 4 °C for 20 min to remove the cells. The supernatant (2 mL) was saturated with NaCl followed by extraction with 2 × 2 mL of HPLC eluent (*n*-hexane/*i*-PrOH = 95/5, *v/v*) by shaking for 5 min. The combined organic layer was dried over Na_2_SO_4_ and measured by HPLC for yield and *ee*.

Resting cells of 48 h-, 72 h- and 96-h-grown ages were also suspended in 10 mL Na_2_HPO_4_-KH_2_PO_4_ buffer (pH 7.0, 100 mM) containing 0.5 g glucose and 10 mM of 1-(3-bromophenyl)ethanone (**1b**). The reaction mixtures were shaken (220 rpm) at 25 °C for 24 h and stopped by centrifugation at 4000 rpm and at 4 °C for 20 min to remove cells. The supernatant (2 mL) was saturated with NaCl followed by extraction with 2 × 2 mL of HPLC eluent (*n*-hexane/*i*-PrOH = 95/5, *v/v*) by shaking for 5 min. The combined organic layer was dried over Na_2_SO_4_ and measured by HPLC for yield and *ee*.

### 4.7. Recyclability

Reactions were carried out with 10 mM substrate **1b** in 10 mL of Na_2_HPO_4_-KH_2_PO_4_ buffer (100 mM, pH 7.0) containing 0.5 g of glucose and 3 g of resting cells of 48-h-grown age, shaken at 25 °C for 24 h. At the end of the reaction, the cells were centrifuged at 4000 rpm for 20 min to be separated from the reaction mixture, then washed by Na_2_HPO_4_-KH_2_PO_4_ buffer (100 mM, pH 7.0) and resuspended in 10 mL of the same buffer containing the same substrates and glucose. The reaction mixture (2 mL of supernatant separated from cells) was saturated with NaCl followed by extraction with 2 × 2 mL of HPLC eluent (*n*-hexane/*i*-PrOH = 95/5, *v/v*) by shaking for 5 min. The combined organic layer was dried over Na_2_SO_4_ and measured by HPLC for yield and *ee*.

### 4.8. General Methods for Resting Cell Biotransformation

Reactions were performed in 50-mL screw-capped glass vials to prevent evaporation of the substrate/product. Shaking was performed in a heated ground-top shaker at 25 °C with 220 rpm. Approximately 3 g of resting cells of 48-h-grown age (wet cells) were resuspended in 10 mL of Na_2_HPO_4_-KH_2_PO_4_ buffer (100 mM, pH 7.0) containing 0.5 g of glucose and 10 mM **1a**–**1n**. After 24 h, the reaction was stopped by centrifugation at 4000 rpm and at 4 °C for 20 min to remove cells. The supernatant (2 mL) was saturated with NaCl followed by extraction with 2 × 2 mL of HPLC eluent (*n*-hexane/*i*-PrOH = 95/5, *v/v*) by shaking for 5 min. The combined organic layer was dried over Na_2_SO_4_ and measured by HPLC for yield and *ee*.

### 4.9. Preparative-Scale Synthesis of Enantiomeric β-Phenylalcohols (S)-***2a***–***2i***, (S)-***2m*** and (S)-***2n*** by Resting Cells

For isolation and characterization of the bioreduction product, the reaction was performed on a preparative scale: 300 g resting cells of *R. rubra* AS 2.2241 were resuspended in 1000 mL of Na_2_HPO_4_-KH_2_PO_4_ buffer (100 mM, pH 7.0) with 50 g glucose and 10 mM of each substrate (**1a**–**1i**, **1m** and **1n**). The reaction mixture was incubated at 25 °C and shaken at 220 rpm for 24 h. The cells were removed by centrifugation and the supernatant was saturated with NaCl. The supernatant was extracted with EtOAc (1000 mL × 3). The organic phases were washed with brine, dried over Na_2_SO_4_, filtered and concentrated in vacuo. The residue was purified by flash chromatography on silica gel (eluent: EtOAc/PE 1:20) to give the enantiomerically pure alcohols **2a**–**2i**, **2m**, **2n**. The isolated yield and *ee* of preparative-scale are comparable to those obtained from screening biotransformations. The spectroscopic data (^1^H and ^13^ C NMR, and HPLC retention times) of enantiomerically alcohols **2a**–**2i**, **2m** and **2n** are in agreement with those obtained for racemic forms, as described in the Supplementary Materials.

### 4.10. Assay Methods

Reaction products were analysed by chiral HPLC analysis using a Shimadzu LC-10AT VP series (Tokyo, Japan) and a Shimadzu SPD-M10Avp photo diode array detector (190–370 nm) with a Chiralcel AD-H column [eluent: *n*-hexane/*i*-PrOH (95:5, *v/v*), flow rate: 0.5 mL/min, column temperature 25 °C]. The yields (quantified using calibration curves) and product *ee* values of analytes were determined by chiral HPLC analyses according to the following retention time data: 1-(2-bromophenyl)ethanone (**1a**) [**1a**, 11.85 min; (*R*)-**2a**, 12.71 min (*S*)-**2a**, 13.23 min], 1-(3-bromophenyl)ethanone (**1b**) [**1b**, 10.66 min; (*S*)-**2b**, 16.39 min; (*S*)-**2b**, 17.26 min], 1-(3-bromophenyl)ethanone (**1c**) [**1c**, 11.21 min; (*S*)-**2c**, 16.83 min; (*R*)-**2c**, 17.99 min], 1-(2-nitrophenyl)ethanone (**1d**) [**1d**, 12.54; (*R*)-**2d**, 21.33 min; (*S*)-**2d**, 22.63 min], 1-(3-nitrophenyl)ethanone (**1e**) [**1e**, 21.14 min; (*R*)-**2e**, 26.25 min; (*S*)-**2e**, 27.06 min], 1-(3-nitrophenyl)ethanone (**1f**) [**1f**, 20.24 min; (*S*)-**2f**, 35.54 min; (*R*)-**2f**, 38.20 min], 1-(2-(trifluoromethyl)phenyl)ethanone (**1g**) [**1g**, 12.49 min; (*R*)-**2g**, 11.83 min; (*S*)-**2g**, 12.34 min], 1-(3-(trifluoromethyl)phenyl)ethanone (**1h**) [**1h**, 11.13 min; (*S*)-**2h**, 14.06 min; (*R*)-**2h**, 15.57 min], 1-(4-(trifluoromethyl)phenyl)ethanone (**1i**) [**1i**, 11.68 min; (*S*)-**2i**, 16.55 min; and (*R*)-**2i**, 17.79 min], 1-(2,4-dichlorophenyl)ethanone (**1m**) [**1m**, 15.09 min; (*R*)-**2m**, 19.09 min; (*S*)-**2m**, 21.26 min], 1-(3,4-dichlorophenyl)ethanone (**1n**) [**1n**, 12.39 min; (*R*)-**2n**, 12.79 min; and (*S*)-**2n**, 13.67 min].

The optical rotations of products isolated from the biocatalytic reactions were determined in a 1-dm cuvette using a Perkin-Elmer model 241 polarimeter and were referenced to the Na–D line. The experimental and reported data are listed below: (*S*)-**2a**, ${\left[\alpha \right]}_{D}^{20}$ = −62.4 (*c* 1.00, CHCl_3_) {(*S*)-1-(2-bromophenyl)ethanol ${\left[\alpha \right]}_{D}^{27}$ = −29.8 (*c* 0.68, CHCl_3_) [42]}; (*S*)-**2b**, ${\left[\alpha \right]}_{D}^{20}$ = −43.9 (*c* 1.00, CHCl_3_) {(*S*)-1-(3-bromophenyl)ethanol ${\left[\alpha \right]}_{D}^{25}$ = −27.6 (*c* 1.00 in CHCl_3_) [35]}; (*S*)-**2c**, ${\left[\alpha \right]}_{D}^{20}$ = −17.3 (*c* 1.00, MeOH, {(*S*)-1-(4-bromophenyl)ethanol ${\left[\alpha \right]}_{D}^{21}$ = −20.6 (*c* 1.07, MeOH) [39]}; (*S*)-**2d**, ${\left[\alpha \right]}_{D}^{20}$ = +64.9 (*c* 0.1, MeOH) {(*S*)-1-(2-nitrophenyl)ethanone ${\left[\alpha \right]}_{D}^{25}$ = +18.5 (*c* 0.23, MeOH) [40]}; (*S*)-**2e**, ${\left[\alpha \right]}_{D}^{20}$ = −88.5 (*c* 0.1, CHCl_3_) {(*S*)-1-(3-nitrophenyl)ethanone ${\left[\alpha \right]}_{D}^{25}$ = −20.5 (*c* 1.0, CHCl_3_) [40]}; (*S*)-**2f**, ${\left[\alpha \right]}_{D}^{20}$ = −67.2 (*c* 0.1, CHCl_3_) {(*S*)-1-(4-nitrophenyl)ethanone ${\left[\alpha \right]}_{D}^{25}$ = −20.5 (*c* 1.2, CHCl_3_) [40]}; (*S*)-**2g**, ${\left[\alpha \right]}_{D}^{20}$ = −43.1 (*c* 0.1, MeOH) {(*S*)-1-(2-Trifluoromethylphenyl) ethanol ${\left[\alpha \right]}_{D}^{26}$ = −37.7 (*c* 1.0, CH_3_OH) [42]}; (*S*)-**2h**, ${\left[\alpha \right]}_{D}^{20}$ = −55.1 (*c* 0.2, MeOH) {(*S*)-1-(3-(trifluoromethyl)phenyl)ethanol ${\left[\alpha \right]}_{D}^{25}$ = −21.9 (*c* 1.40, MeOH) [35]}; (*S*)-**2i**, ${\left[\alpha \right]}_{D}^{20}$ = −62.2 (*c* 0.2, CHCl_3_) {(*S*)-1-(4-(trifluoromethyl)phenyl)ethanol ${\left[\alpha \right]}_{D}^{25}$ = −33.7 (*c* 5.52, CHCl_3_) [35]}; (*S*)-**2m**, ${\left[\alpha \right]}_{D}^{20}$ = −75.05 (*c* 0.2, CHCl_3_) {(*S*)-1-(2,4-dichlorophenyl)ethanol ${\left[\alpha \right]}_{D}^{25}$ = −52.4 (*c* 0.55, CHCl_3_) [43]}; (*S*)-**2n**, ${\left[\alpha \right]}_{D}^{20}$ = −30.0 (*c* 0.3, CHCl_3_) {(*R*)-1-(3,4-dichlorophenyl)ethanol ${\left[\alpha \right]}_{D}^{20}$ = +35.8 (*c* 1.00, CHCl_3_) [41]}.

## Figures and Tables

**Table 1:** Bioreduction of 1-(3-bromophenyl)ethan-1-one (**1b**) by marine-derived fungi.

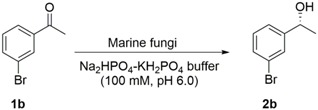				
Entry	Strains	Yield (%) ^[a]^	*Ee* (%) ^[a]^	Genome Sequences Available in Our Laboratory
1	*P. citrinum* GIM 3.458	25.3	99 (*S*)	No
2	*P. citrinum* GIM 3.251	5.5	53.7 (*S*)	No
3	*P. citrinum* GIM 3.100	39.2	99 (*S*)	No
4	*A. sclerotiorum* AS 3.2578	n.d.	n.d.	No
5	*A. sydowii* AS 3.7839	n.d.	n.d.	No
6	*A. sydowii* AS 3.6412	n.d.	n.d.	No
7	*G. candidum* GIM 2.361	75.9	99 (*S*)	No
8	*G. candidum* GIM 2.616	75.1	99 (*S*)	No
9	*R. rubra* GIM 2.31	99	99 (*S*)	No
10	*R. mucilaginosa* GIM 2.157	82	99 (*S*)	No
11	*G. candidum* AS 2.1183	44.3	99 (*S*)	No
12	*G. candidum* AS 2.498	77.7	99 (*S*)	No
13	*R. rubra* AS 2.2241	99	99 (*S*)	Yes ^[b]^
control	no	n.d.	n.d.	-

^[a]^ Yield and *ee* values were determined by HPLC equipped with a Chiracel OD-H chiral column; ^[b]^ Genome sequences were available in our laboratory, of which the genomic DNA libraries for the Illumina platform were generated and sequenced at BGI (Shenzhen, China). n.d. = not determined.

**Table 2:** Reduction of **1b** with growing cells of *R. rubra* AS 2.2241 in RM1 medium ^[a]^.

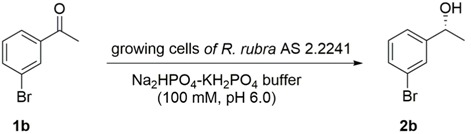						
Entry	1b at 0 h (mM)	2b at 24 h (mM) ^[b]^	2b at 48 h (mM) ^[b]^	2b at 72 h (mM) ^[b]^	2b at 96 h (mM) ^[b]^	Cell Mass (Dry wt., g/L) ^[c]^
1	5	2.36 (*ee*_S_ = 99)	3.06 (*ee*_S_ = 99)	4.95 (*ee*_S_ = 99)	4.07 (*ee*_S_ = 99)	4.61
2	10	0.42 (*ee*_S_ = 99)	8.33 (*ee*_S_ = 99)	9.9 (*ee*_S_ = 99)	6.21 (*ee*_S_ = 99)	2.81
3	11.6	n.c.	0.93 (*ee*_S_ = 99)	8.41 (*ee*_S_ = 99)	4.96 (*ee*_S_ = 99)	1.12
4	13.3	n.c.	n.c.	n.c.	n.c.	0.46
5	15	n.c.	n.c.	n.c.	n.c.	0
Control						
1′	10 (without inoculation) ^[d]^	0	0	0	0	0
2′	0 (blank) ^[e]^	0	0	0	0	7.16
3′	10 (with 5 g dry dead cells) ^[f]^	0	0	0	0	n.d.

^[a]^ 200 mL of RM1 medium, one loop of a single colony of *R. rubra* AS 2.2241, given amount of **1b**, cultured at 28 °C, 220 rpm for given times; ^[b]^ concentrations of **2b** were determined by HPLC analysis equipped with a Chiracel OD-H chiral column; ^[c]^ The cells were harvested by centrifugation at 4000 rpm and at 4 °C for 20 min; ^[d]^ This was used as a positive control for 1-(3-bromophenyl)ethan-1-one (**1b**); ^[e]^ This was done to check the growth of *R. rubra* AS 2.2241 in the absence of **1b**; ^[f]^ This was used to determine active enzymes by the cells; n.c. = no conversion; n.d. = not determined.

**Table 3:** Stereoselective reduction of prochiral ketones with growing cells of *R. rubra* AS 2.2241 ^[a]^.

Entry	Substrates	Yield (%) ^[b]^	*Ee* (%) ^[b]^	Config. ^[c]^
1	**1a**	99	99	*S*
2	**1b**	99	99	*S*
3	**1c**	99	99	*S*
4	**1d**	99	99	*S*
5	**1e**	99	99	S
6	**1f**	99	99	*S*
7	**1g**	99	99	*S*
8	**1h**	99	99	*S*
9	**1i**	99	99	*S*
10	**1j**	n.c.	n.c.	n.d.
11	**1k**	n.c.	n.c.	n.d.
12	**1l**	n.c.	n.c.	n.d.
13	**1m**	n.c.	n.c.	n.d.
14	**1n**	n.c.	n.c.	n.d.

^[a]^ Reaction conditions: 200 mL of RM1 medium, one loop of a single colony of *R. rubra* AS 2.2241, 10 mM of substrates **1a**–**1n**, inoculated at 28 °C, 220 rpm for 72 h; ^[b]^ Yield and *ee* were determined by chiral HPLC analysis equipped with a Chiracel OD-H chiral column; ^[c]^ The absolute configurations of the reduction products were determined by comparing the measured specific signs of rotation with those reported in the literature [35,39,40,41,42,43]; n.c. = no conversion; n.d. = not determined.

**Table 4:** Reduction of **1b** with resting cells of *R. rubra* AS 2.2241 of different ages ^[a]^.

Entry	Types of Resting Cells	Reaction Conditions	Yield of 2b (%) ^[b]^	*Ee* of 2b (%) ^[b]^
1	24-h-grown	25 °C, pH 6, 24 h	59	99 (*S*)
2		25 °C, pH 7, 24 h	89	99 (*S*)
3		25 °C, pH 8, 24 h	52	99 (*S*)
4		20 °C, pH 7, 24 h	63	99 (*S*)
5		35 °C, pH 7, 24 h	41	99 (*S*)
6	48-h-grown	25 °C, pH 7, 24 h	99	99 (*S*)
7	72-h-grown	25 °C, pH 7, 24 h	81	99 (*S*)
8	96-h-grown	25 °C, pH 7, 24 h	77	99 (*S*)

^[a]^ Reaction conditions: 3 g resting cells of *R. rubra* AS 2.224 of required ages, 10 mM of substrates **1b**, and 0.5 g glucose in 10 mL Na_2_HPO_4_-KH_2_PO_4_ buffer (100 mM, given pH-values), inoculated at given temperatures, 220 rpm for 24 h; ^[b]^ Yield and *ee* were determined using chiral HPLC instrument equipped with a Chiracel OD-H chiral column; ^[c]^ The absolute configurations of the reduction products were determined by comparing the specific signs of rotation measured with those reported in the literature [35,39,40,41,42,43].

**Table 5:** Stereoselective reduction of prochiral ketones with resting cells of *R. rubra* AS 2.2241 ^[a]^.

Entry	Substrates	Yield (%) ^[b]^	*Ee* (%) ^[b]^	Config. ^[c]^
1	**1a**	99	99	*S*
2	**1b**	99	99	*S*
3	**1c**	99	99	*S*
4	**1d**	99	99	*S*
5	**1e**	99	99	*S*
6	**1f**	99	99	*S*
7	**1g**	99	99	*S*
8	**1h**	99	99	*S*
9	**1i**	99	99	*S*
10	**1j**	n.c.	n.c.	n.d.
11	**1k**	n.c.	n.c.	n.d.
12	**1l**	n.c.	n.c.	n.d.
13	**1m**	63	28	*S*
14	**1n**	55	99	*S*

^[a]^ Reaction conditions: 10 mL Na_2_HPO_4_-KH_2_PO_4_ buffer (100 mM, pH 7.0), 3 g wet cells of 48-h-grown age, 10 mM various aromatic ketones, 0.5 g glucose, 25 °C, 24 h; ^[b]^ Yield and *ee* were determined by chiral HPLC instrument equipped with a Chiracel OD-H chiral column; ^[c]^ The absolute configurations of the reduction products were determined by comparing the specific signs of rotation measured with those reported in the literature [35,39,40,41,42,43]; n.c. = no conversion; n.d. = not determined.

## Supplementary Materials

The following are available online at http://www.mdpi.com/1660-3397/16/2/62/s1. The NMR spectra and HPLC spectra of all the compounds are shown.
