# Supplementary material for: A Comparative Study on Asymmetric Reduction of Ketones Using the Growing and Resting Cells of Marine-Derived Fungi
Source: Mar Drugs. 2018 Feb 14;16(2):62. doi: 10.3390/md16020062 (PMC5852490; doi:10.3390/md16020062)
Supplement: Supplementary file 1 [file marinedrugs-16-00062-s001.pdf]

Supplementary Material

A comparative study on asymmetric reduction of ketones using the growing and resting cells of marine-derived fungi

Hui Liu[1], Bi-Shuang Chen[1,2,3]*, Fayene Zeferino Ribeiro de Souza[4], Lan Liu[1,2,3]

[1] School of Marine Sciences, Sun Yat-Sen University, Guangzhou 510275, People's Republic of China; liuh229@mail.sysu.edu.cn (H. L.); cesllan@mail.sysu.edu.cn (L. L.)
[2] Guangdong Provincial Key Laboratory of Marine Resources and Coastal Engineering, Guangzhou 510275, China
[3] South China Sea Bio-Resource Exploitation and Utilization Collaborative Innovation Center, Sun Yat-Sen University, Guangzhou, 510275, China
[4] Departamento de Química, Faculdade de Ciências, UNESP, Bauru 17033-360, Brazil
* Correspondence: chenbsh23@mail.sysu.edu.cn; Tel.: +86-20-84725459

Contents:

NMR spectra of racemic alcohols **2a-2f**, **2m** and **2n**……………………………………………………2
Chiral HPLC spectra of enantiomerically alcohols (*S*)-**2a-2i**, (*S*)-**2m** and (*S*)-**2n**……………………...10

^{1}H-NMR (**2a**)

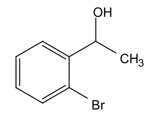

1-(2-bromophenyl)ethanol

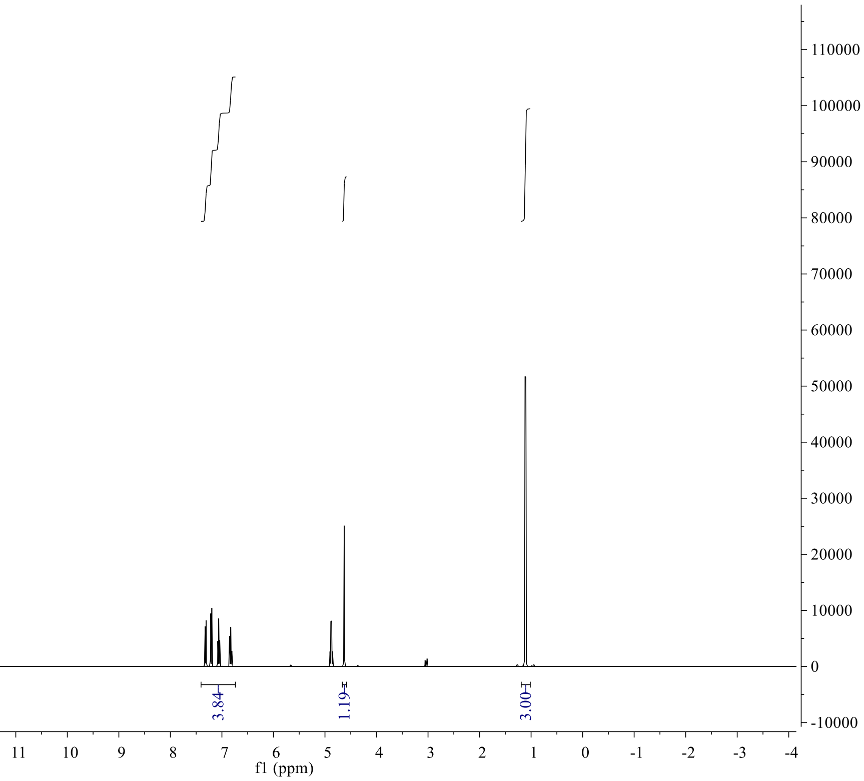

^{13}C-NMR (**2a**)

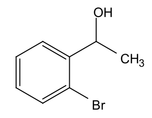

1-(2-bromophenyl)ethanol

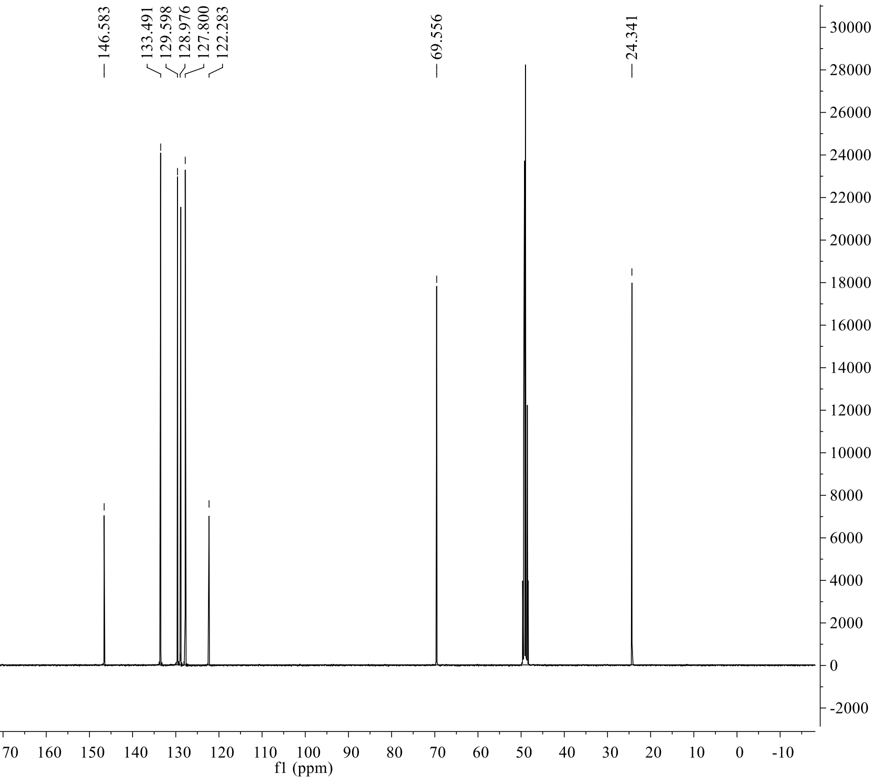

^{1}H-NMR (**2b**)

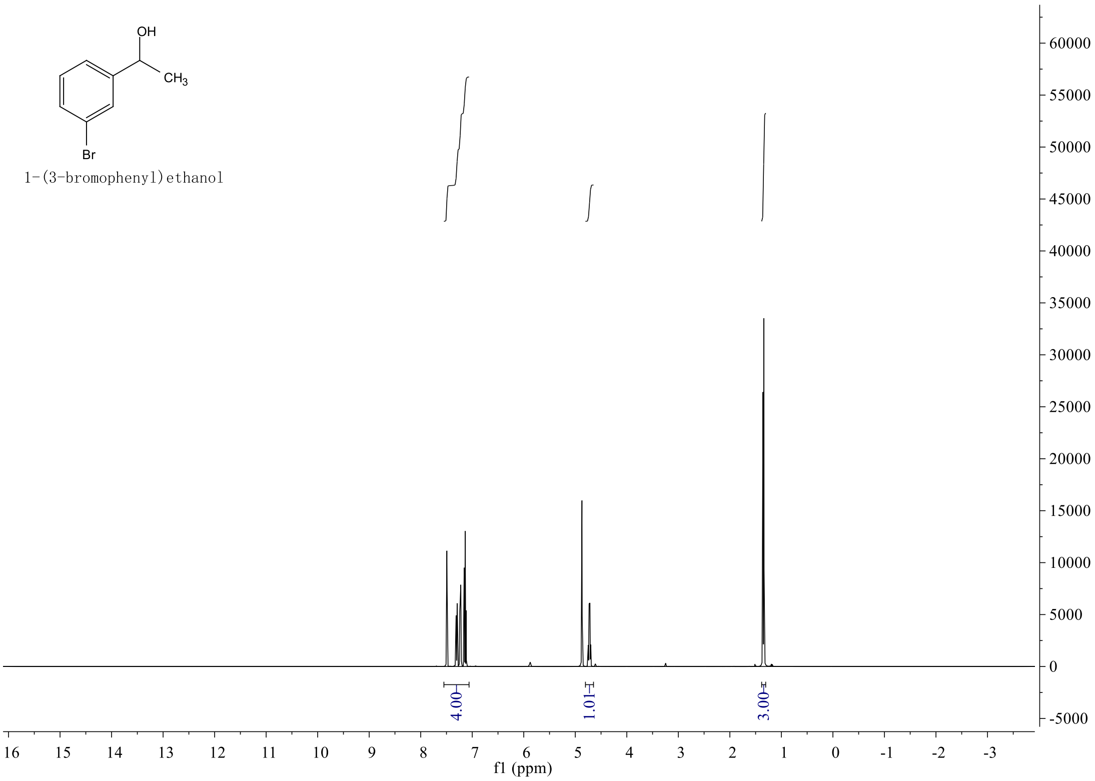

^{13}C-NMR (**2b**)

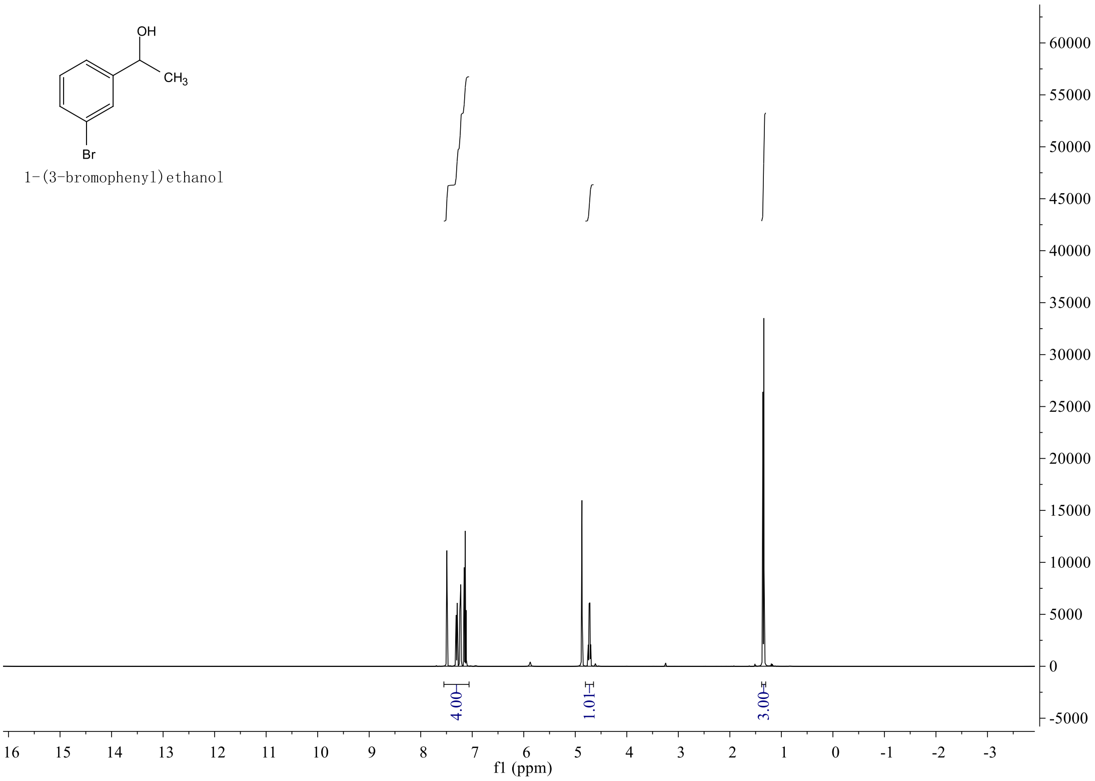

^{1}H-NMR (**2c**)

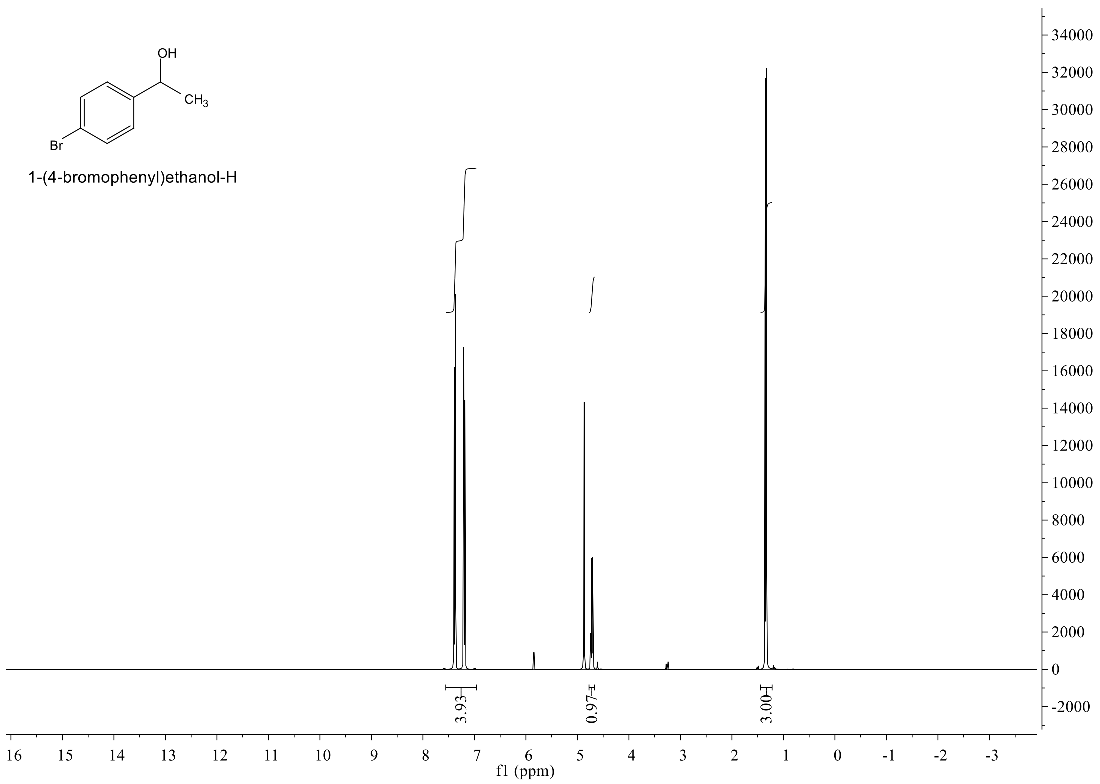

^{13}C-NMR (**2c**)

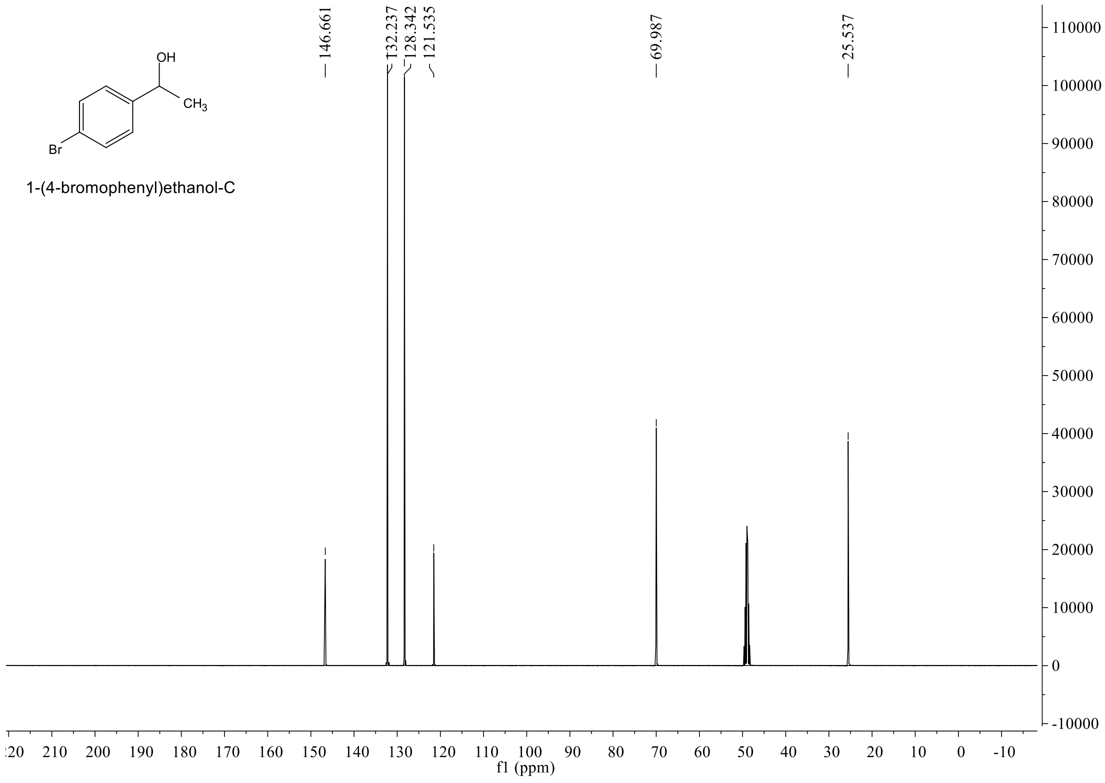

^{1}H-NMR (**2d**)

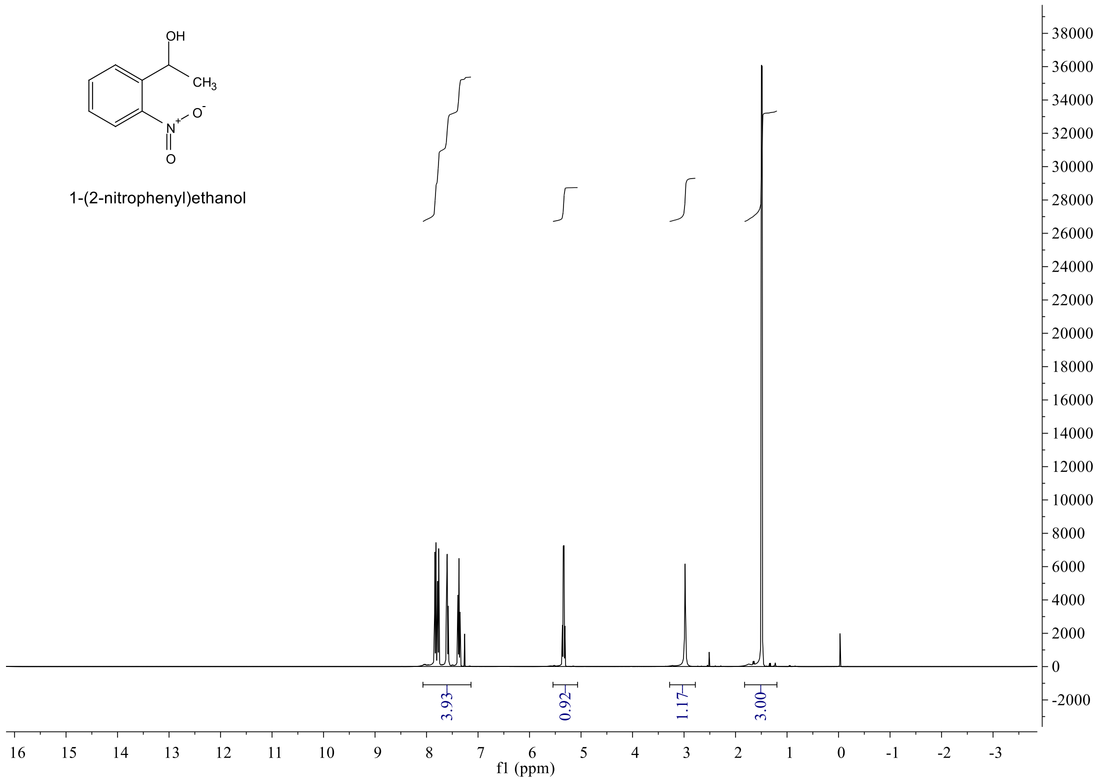

^{13}C-NMR (**2d**)

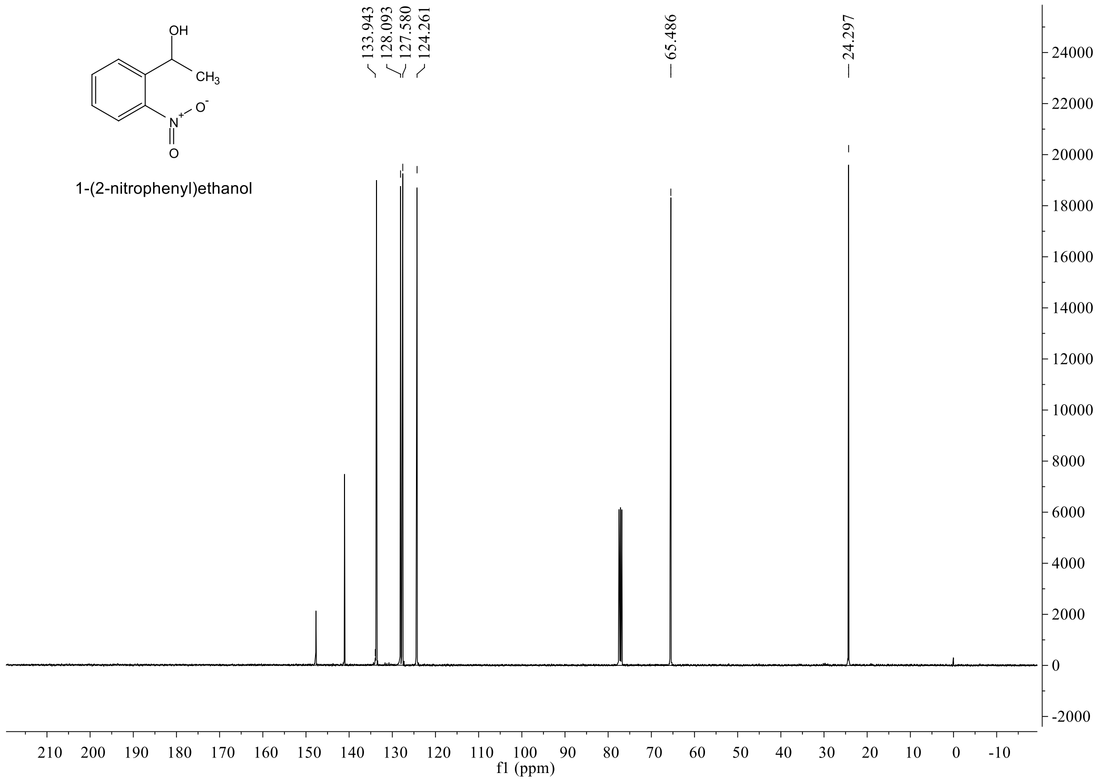

^{1}H-NMR (**2e**)

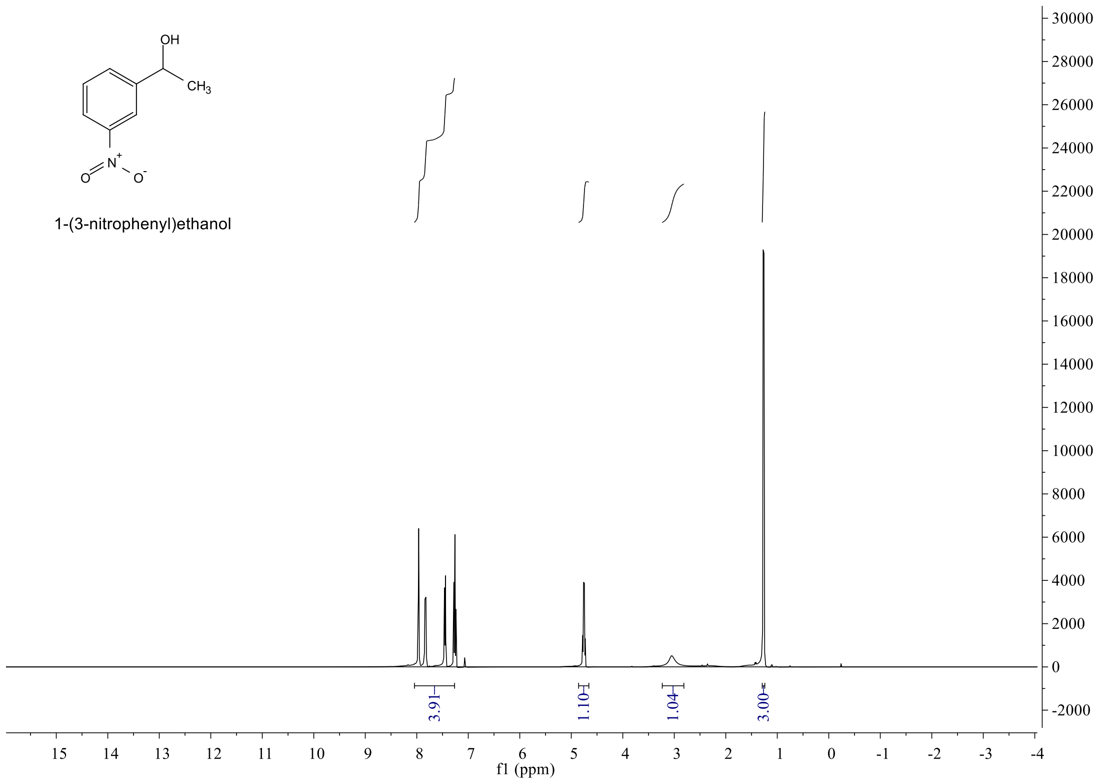

^{13}C-NMR (**2e**)

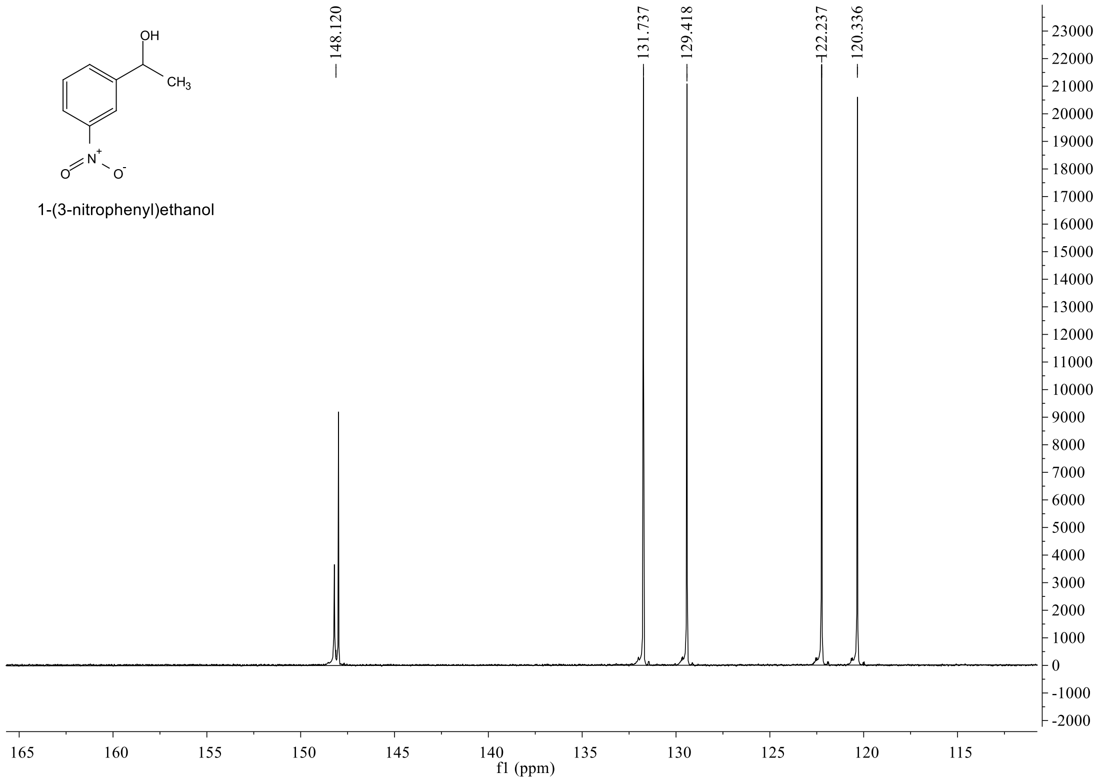

^{1}H-NMR (**2f**)

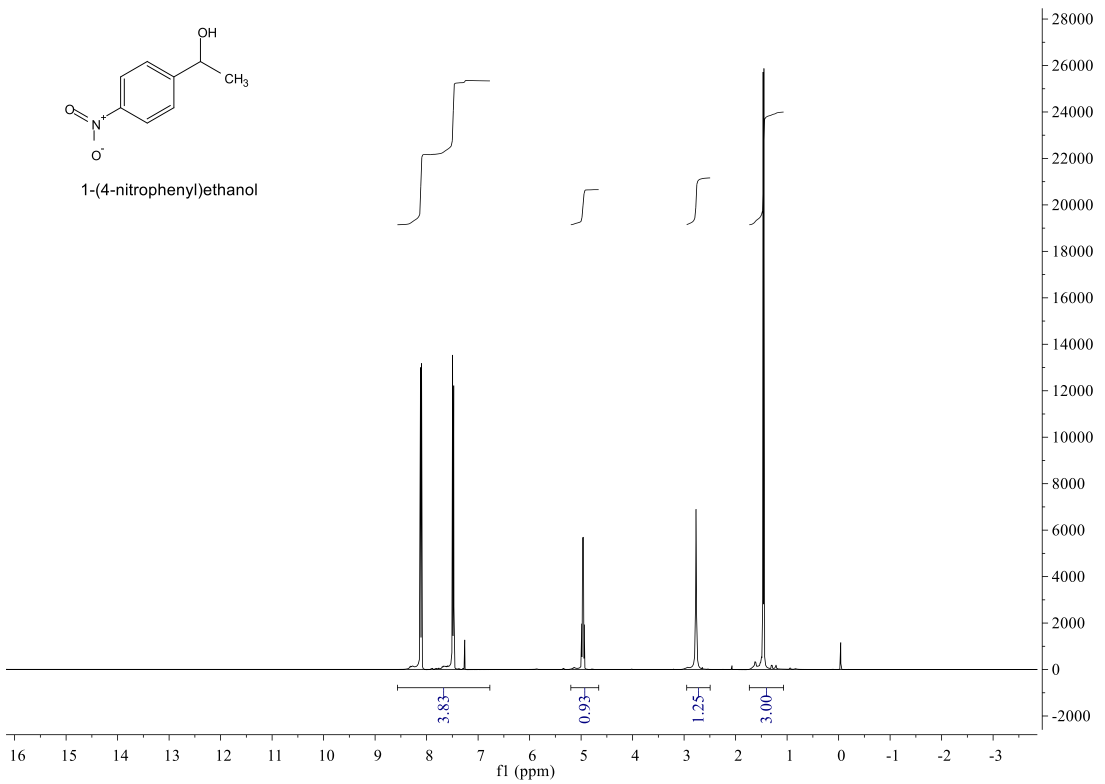

^{13}C-NMR (**2f**)

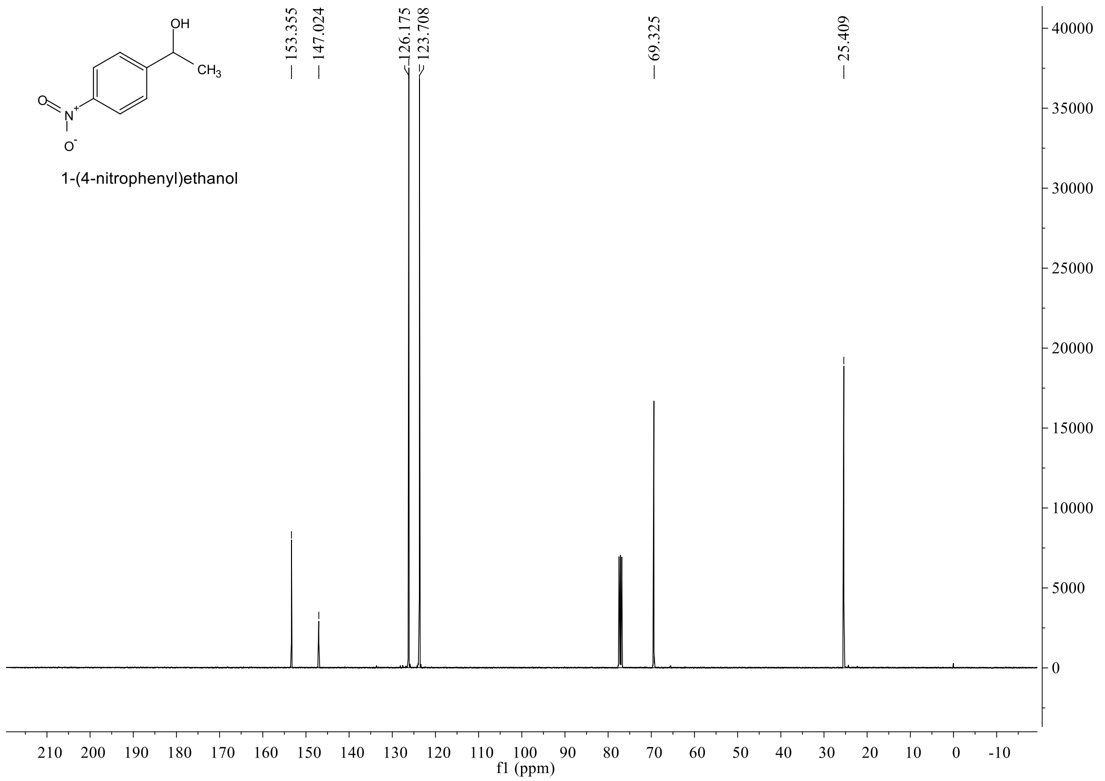

^{1}H-NMR (**2m**)

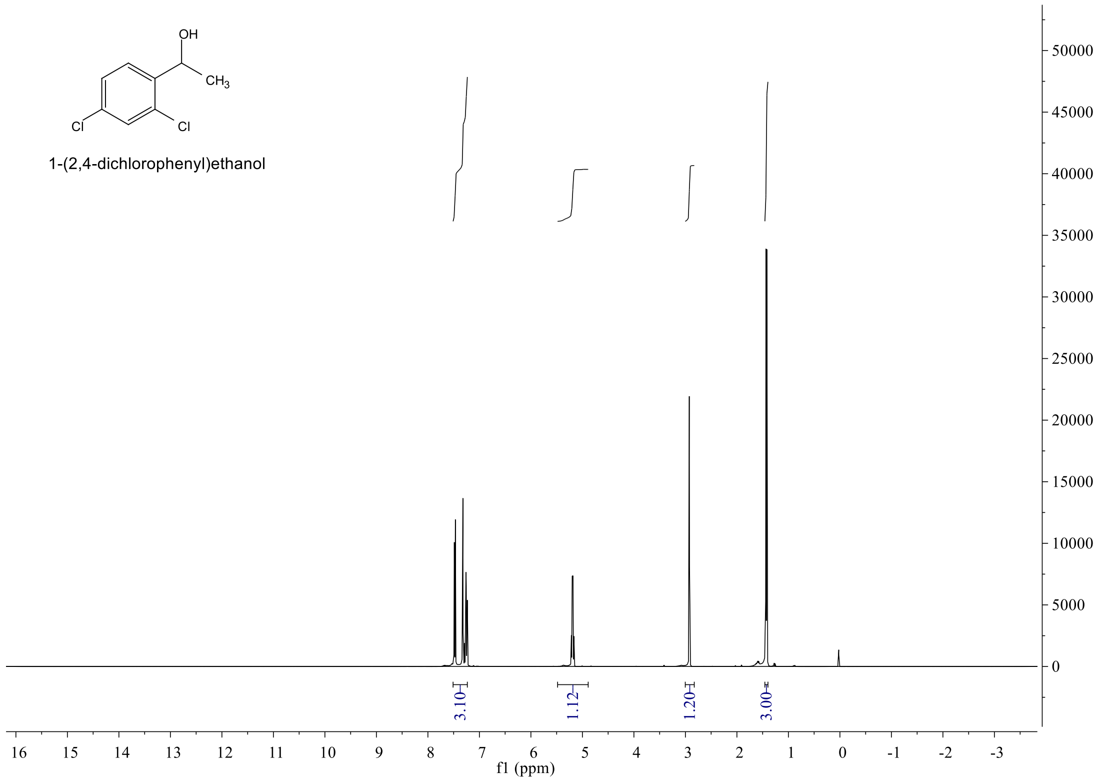

^{13}C-NMR (**2m**)

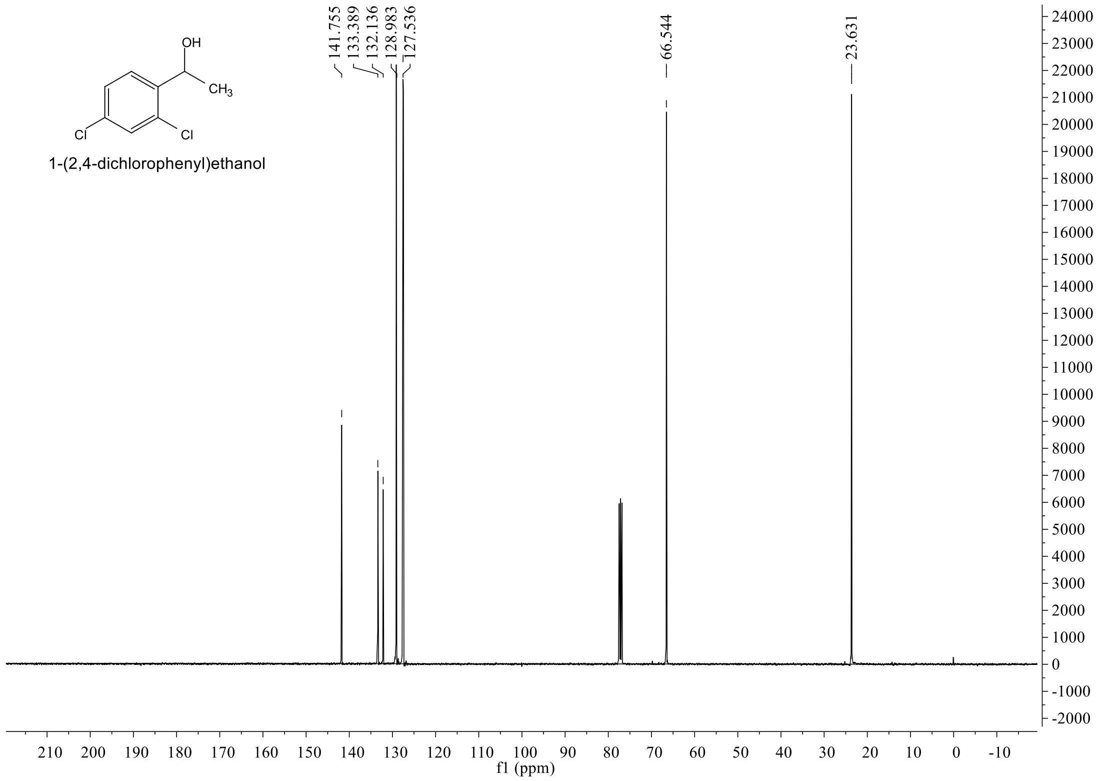

^{1}H-NMR (**2n**)

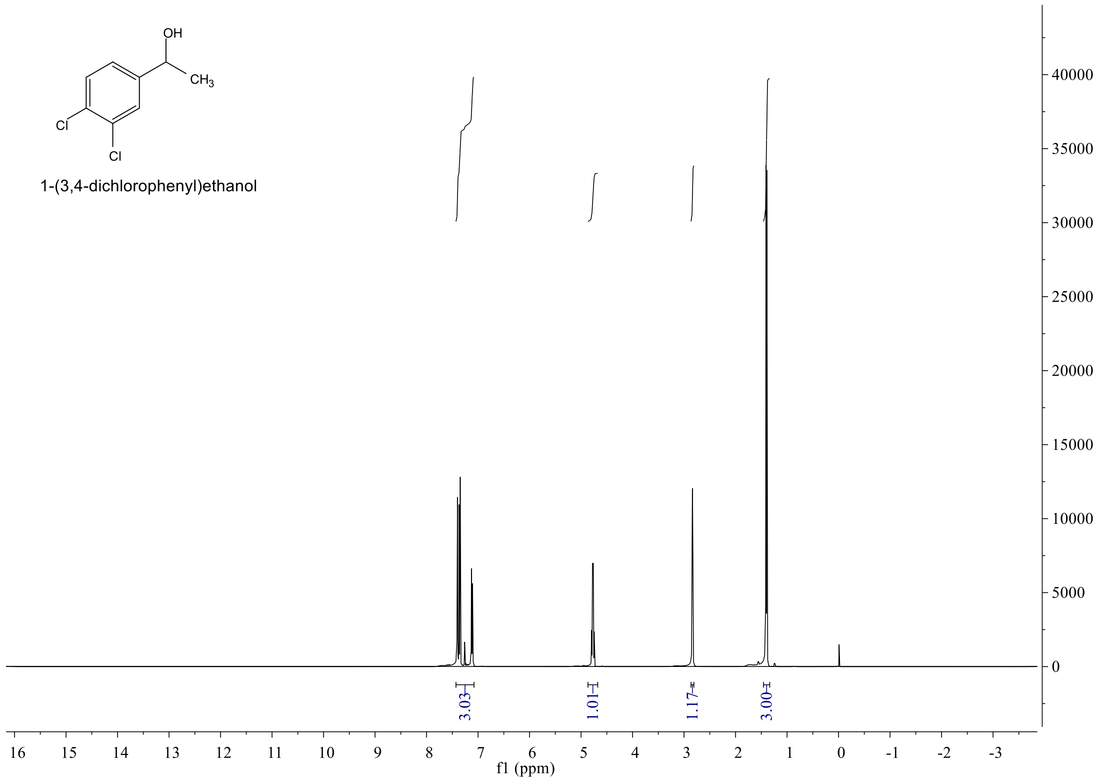

^{13}C-NMR (**2n**)

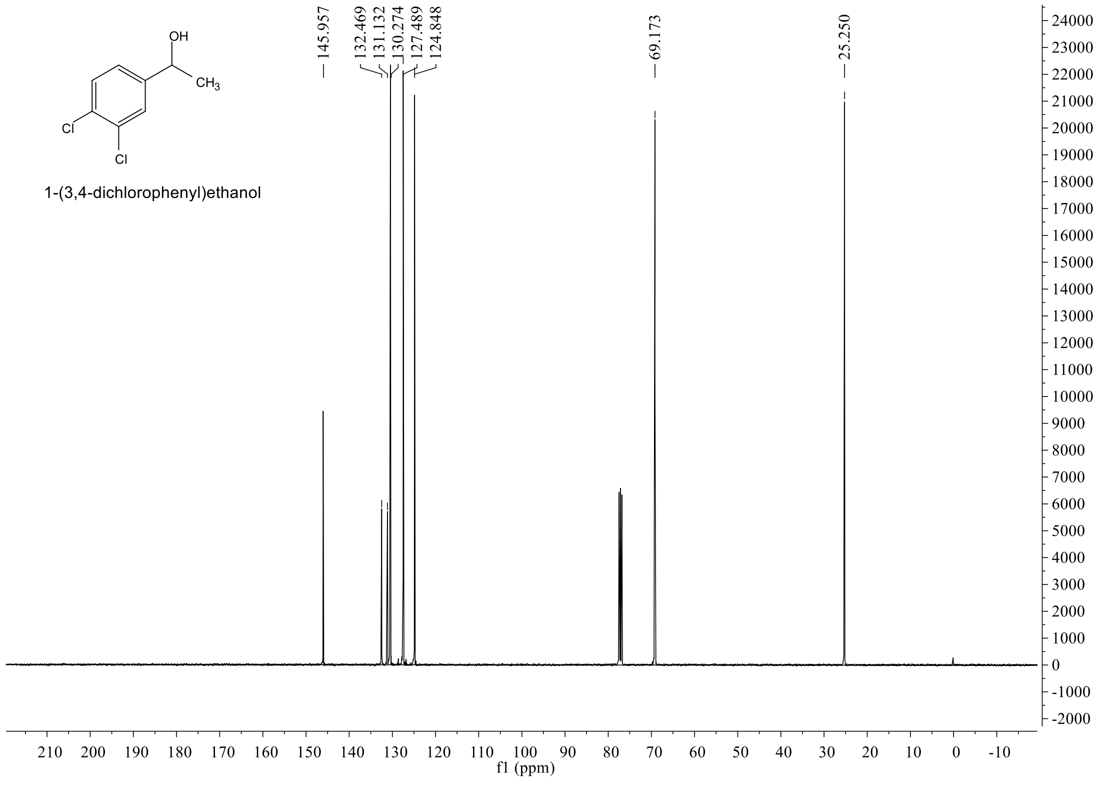

Rac-**2a**

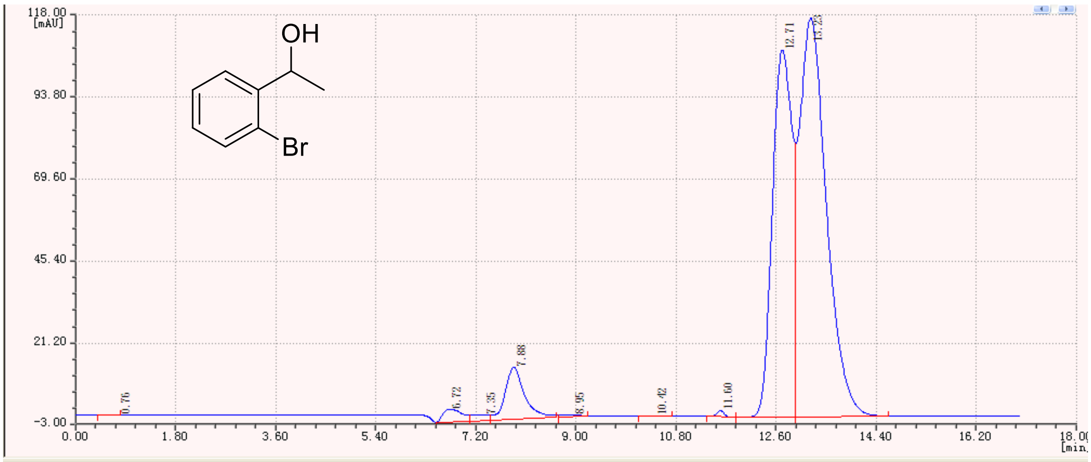

参数设置	分析结果	分组结果	样品信息						
ID	组 分 名	RT (分钟)	峰 高	峰 面 积	浓 度	半峰宽 (秒)	分离度	理论塔板数	拖尾因子
1		0.760	4	56.0	0.0078	13.17	0.00	66.41	0.56
2		6.715	366	9045.1	1.2613	23.23	11.55	1665.37	1.45
3		7.352	170	3688.0	0.5143	20.39	1.03	2590.51	0.74
4		7.882	1533	37185.9	5.1856	22.80	0.87	2381.46	1.45
5		8.945	40	886.1	0.1236	20.82	1.72	3678.01	1.03
6		10.422	10	197.0	0.0275	18.52	2.65	6311.96	0.86
7		11.603	185	1669.6	0.2328	8.48	3.09	37286.03	0.78
8		12.714	10837	269770.2	37.6196	23.40	2.46	5883.45	0.75
9		13.227	11763	394602.1	55.0275	31.53	0.66	3506.69	2.51
		总计:	Σ=24908	Σ=717100.0	Σ=100....				

(*S*)-**2a**

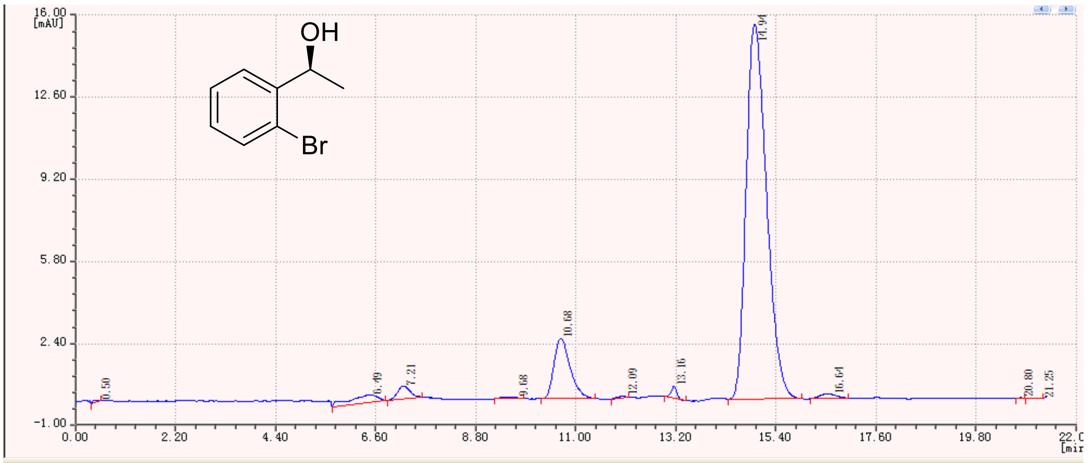

参数设置	分析结果	分组结果	样品信息						
ID	组 分 名	RT (分钟)	峰 高	峰 面 积	浓 度	半峰宽 (秒)	分离度	理论塔板数	拖尾因子
1		0.502	10	61.2	0.1051	5.75	0.00	151.46	0.73
2		6.488	31	1440.8	2.4743	43.69	8.55	439.56	0.70
3		7.208	53	1028.9	1.7669	18.25	0.82	3109.96	1.34
4		9.685	7	162.2	0.2785	21.78	4.37	3941.16	0.71
5		10.677	248	6116.5	10.5038	23.18	1.56	4226.91	1.42
6		12.087	5	87.4	0.1502	16.44	2.51	10772.68	0.67
7		13.162	48	405.4	0.6961	7.94	3.11	54785.10	0.79
8		14.937	1551	48423.3	83.1567	29.35	3.36	5162.90	1.42

Rac-**2b**

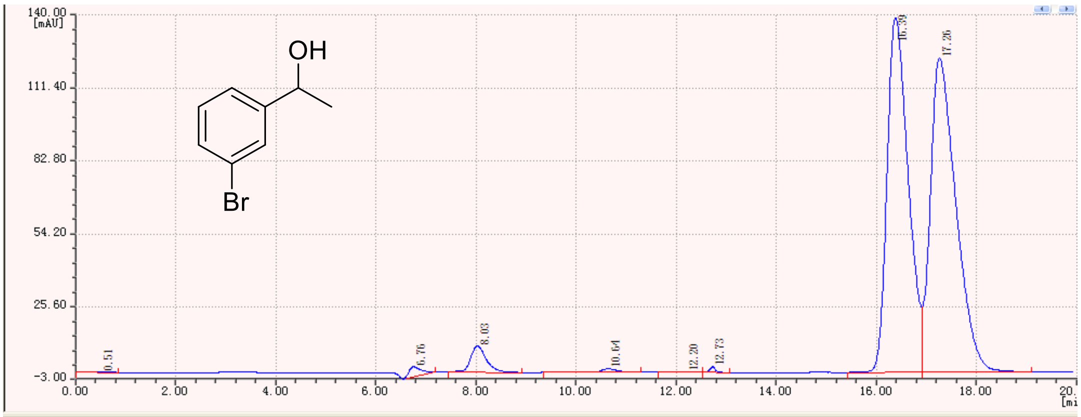

参数设置 分析结果 分组结果 样品信息

ID	组 分 名	RT(分钟)	峰 高	峰 面 积	浓 度	半峰宽(秒)	分离度	理论塔板数	拖尾因子
1		0.510	16	474.6	0.0541	27.88	0.00	6.67	0.77
2		6.758	389	6538.6	0.7447	15.80	10.10	3646.48	1.88
3		8.032	1022	21648.1	2.4657	19.91	2.52	3243.04	0.98
4		10.643	134	3270.1	0.3725	22.94	4.30	4290.64	0.20
5		12.197	29	1002.8	0.1142	32.50	1.98	2806.30	0.80
6		12.733	229	1668.3	0.1900	6.85	0.96	68908.52	0.99
7		16.387	13906	407099.5	46.3681	27.52	7.50	7067.34	1.04
8		17.263	12306	436271.4	49.6907	33.32	1.02	5348.54	2.32
		总计:	Σ=28031	Σ=877973.4	Σ=100....				

(*S*)-**2b**

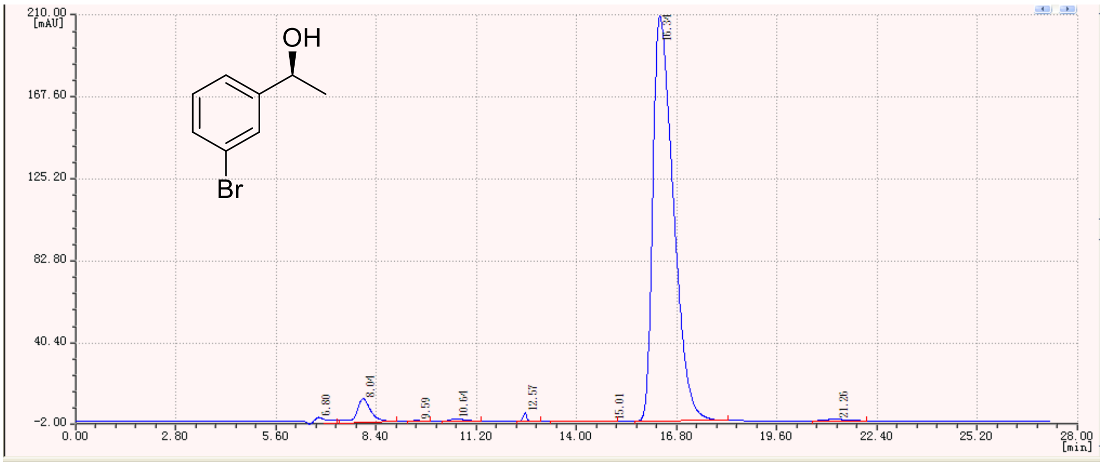

参数设置 分析结果 分组结果 样品信息

ID	组 分 名	RT(分钟)	峰 高	峰 面 积	浓 度	半峰宽(秒)	分离度	理论塔板数	拖尾因子
1		6.799	326	8364.4	0.9418	24.12	0.00	1583.76	1.65
2		8.044	1211	32940.1	3.7089	25.57	1.77	1972.54	1.20
3		9.592	17	342.8	0.0386	18.96	2.45	5103.10	1.06
4		10.642	125	2930.4	0.3299	22.04	1.81	4648.18	1.41
5		12.567	422	2933.8	0.3303	6.53	4.76	73705.04	0.83
6		15.007	25	1826.8	0.2057	68.69	2.29	951.40	0.56
7		16.340	20939	835415.5	94.0641	37.50	0.89	3783.55	1.88

Rac-**2c**

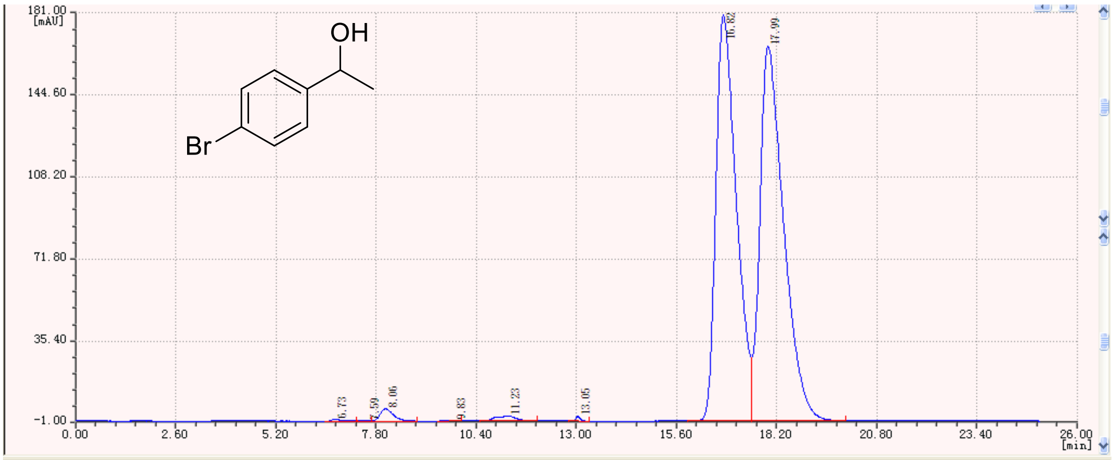

ID	組 分 名	RT (分钟)	峰 高	峰 面 积	浓 度	半峰宽 (秒)	分离度	理论塔板数	拖尾因子
1		6.732	92	2648.8	0.1890	27.06	0.00	1233.13	1.74
2		7.587	44	834.5	0.0596	17.83	1.34	3609.10	0.70
3		8.057	555	13382.6	0.9551	22.67	0.82	2518.25	1.62
4		9.835	11	222.0	0.0158	18.97	3.01	5357.78	0.73
5		11.235	211	8081.5	0.5768	36.00	1.80	1940.85	1.09
6		13.048	216	1627.5	0.1162	7.08	2.97	67644.99	0.83
7		16.825	17997	663657.0	47.3640	34.66	6.39	4695.61	1.17
8		17.987	16609	710731.7	50.7236	40.22	1.10	3985.18	2.17

(*S*)-**2c**

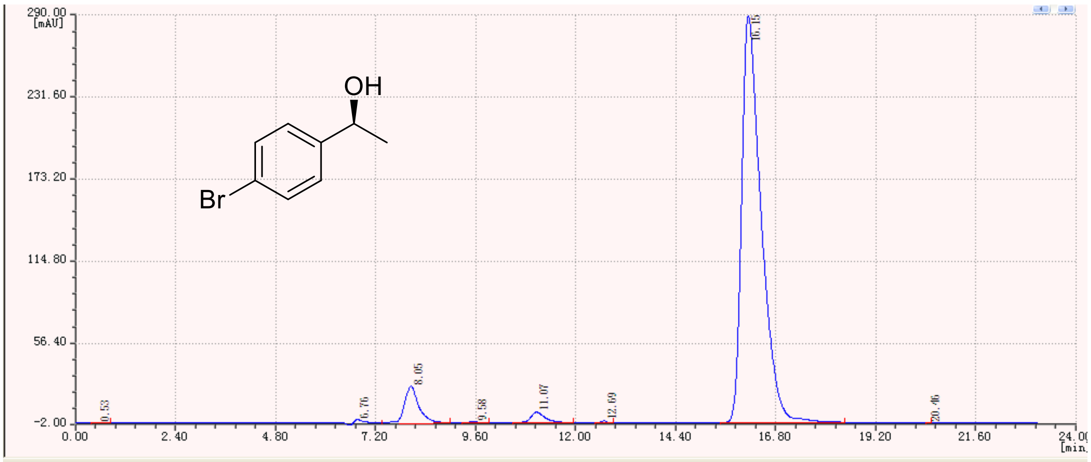

ID	組 分 名	RT (分钟)	峰 高	峰 面 积	浓 度	半峰宽 (秒)	分离度	理论塔板数	拖尾因子
1		0.527	6	78.9	0.0076	12.36	0.00	36.15	1.06
2		6.765	391	9289.8	0.8977	22.33	12.69	1828.62	2.31
3		8.052	2642	64317.9	6.2149	22.88	2.01	2467.35	1.18
4		9.575	24	481.8	0.0466	18.87	2.58	5131.78	1.15
5		11.067	727	16878.1	1.6309	21.82	2.59	5125.36	1.21
6		12.688	128	964.6	0.0932	7.08	3.96	63945.65	0.77
7		16.148	28919	942870.8	91.1077	30.65	6.47	5533.23	2.22

Rac-**2d**

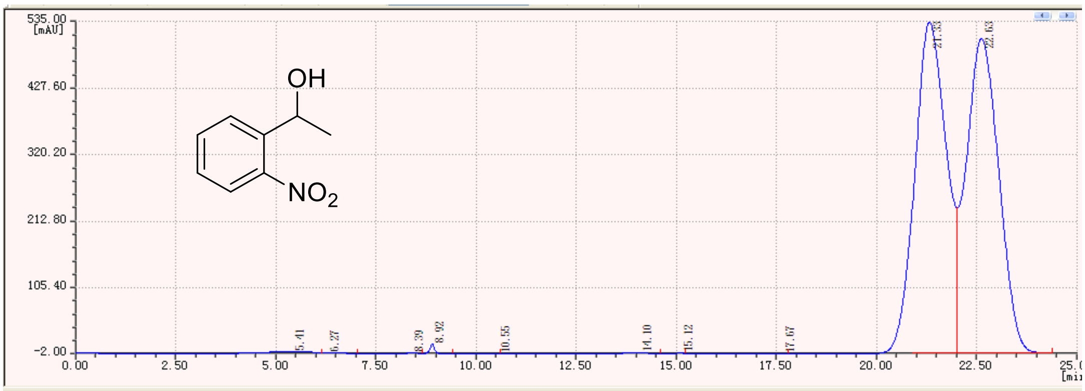

参数设置	分析结果	分组结果	样品信息					

ID	组 分 名	RT(分钟)	峰 高	峰 面 积	浓 度	半峰宽(秒)	分离度	理论塔板数	拖尾因子
1		5.412	317	25341.5	0.4330	75.15	0.00	103.38	0.71
2		6.274	153	4322.2	0.0738	26.55	0.60	1112.46	3.50
3		8.392	43	873.3	0.0149	19.09	3.28	3851.35	0.91
4		8.917	1587	12574.7	0.2148	7.45	1.40	28566.71	0.89
5		10.545	10	1140.6	0.0195	107.22	1.00	192.80	0.62
6		14.095	45	2088.4	0.0357	43.62	1.66	2080.74	0.87
7		15.117	8	152.6	0.0026	17.93	1.17	14169.86	0.75
8		17.670	8	218.0	0.0037	25.61	4.14	9489.02	0.72
9		21.327	53471	2947640.1	50.3597	51.82	3.33	3376.13	0.82
10		22.630	50803	2858825.0	48.8423	52.90	0.88	3648.04	1.55
		总计:	Σ=106445	Σ=5853176.3	Σ=100....				

(*S*)-**2d**

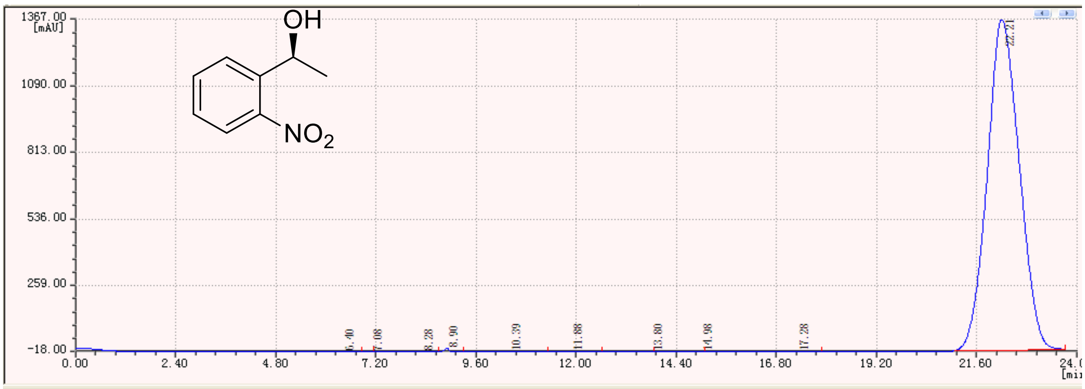

参数设置	分析结果	分组结果	样品信息					

ID	组 分 名	RT(分钟)	峰 高	峰 面 积	浓 度	半峰宽(秒)	分离度	理论塔板数	拖尾因子
1		6.398	130	2215.0	0.0298	16.02	0.00	3180.59	0.93
2		7.075	4	26.8	0.0004	6.31	2.14	25068.95	1.14
3		8.282	157	3529.7	0.0476	21.13	3.10	3060.71	1.09
4		8.897	1591	12064.3	0.1625	7.13	1.54	31049.76	0.92
5		10.387	294	19945.9	0.2687	63.77	1.48	528.70	1.04
6		11.877	178	7042.3	0.0949	37.19	1.04	2032.65	1.18
7		13.802	4	53.1	0.0007	12.47	2.74	24424.30	0.97
8		14.982	5	77.5	0.0010	14.57	3.08	21066.38	1.00
9		17.285	55	1698.5	0.0229	29.03	3.73	7066.79	1.24
10		22.212	137778	7375864.6	99.3715	50.32	4.38	3883.00	1.18
		总计:	Σ=140196	Σ=7422517.6	Σ=100....				

Rac-**2e**

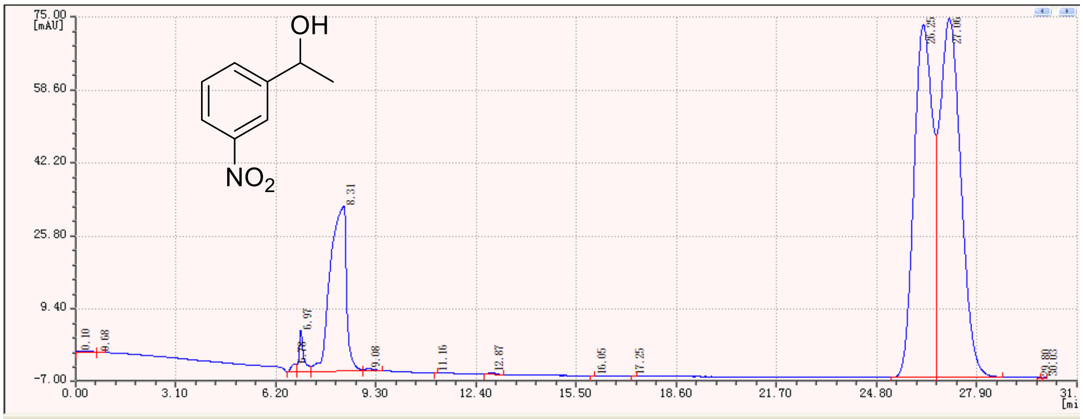

参数设置 分析结果 分組结果 样品信息

ID	組 分 名	RT (分钟)	峰 高	峰 面 积	浓 度	半峰宽 (秒)	分离度	理论塔板数	拖尾因子
2		0.675	10	77.4	0.0094	7.27	1.29	171.71	3.41
3		6.782	174	1952.2	0.2360	10.55	24.19	8240.94	0.65
4		6.967	920	8895.8	1.0754	9.09	0.67	11709.19	1.86
5		8.308	3718	129172.8	15.6161	32.66	2.27	1289.95	0.78
6		9.080	42	886.1	0.1071	19.83	1.04	4177.77	1.59
7		11.158	11	23.7	0.0029	2.03	6.71	604360.57	0.97
8		12.873	35	494.9	0.0598	13.29	7.90	18692.07	0.94
9		16.055	1	10.8	0.0013	10.15	9.58	49878.98	0.63
[illegible]		[illegible]	[illegible]	[illegible]	[illegible]	[illegible]	[illegible]	[illegible]	[illegible]
11		26.245	7928	318161.2	38.4635	37.72	14.68	9647.09	0.76
12		27.060	8071	366664.9	44.3272	42.70	0.72	8002.72	2.02

(*S*)-**2e**

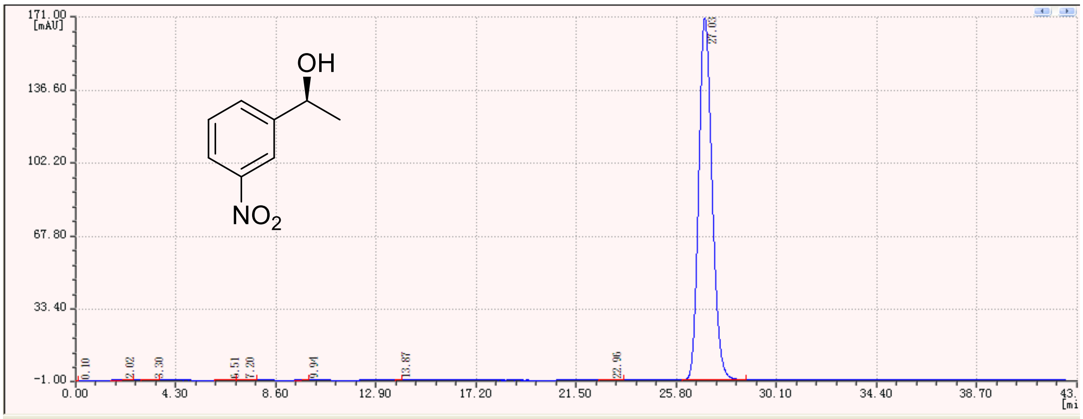

参数设置 分析结果 分組结果 样品信息

ID	組 分 名	RT (分钟)	峰 高	峰 面 积	浓 度	半峰宽 (秒)	分离度	理论塔板数	拖尾因子
1		0.100	4	21.6	0.0032	5.08	0.00	7.71	0.82
2		2.020	36	1115.2	0.1624	29.12	3.96	95.92	1.03
3		3.297	27	723.6	0.1054	25.19	1.66	341.30	0.89
4		6.510	32	928.1	0.1351	27.26	4.32	1136.42	0.87
5		7.203	40	895.3	0.1304	21.04	1.01	2336.06	1.58
6		9.943	12	247.2	0.0360	19.36	4.79	5255.91	0.57
7		13.870	18	155.0	0.0226	8.10	10.09	58499.71	0.96
8		22.962	34	1044.3	0.1521	28.87	17.36	12605.57	1.13
9		27.033	17081	681680.6	99.2530	37.51	4.33	10349.82	1.29
		总计:	Σ=17284	Σ=686811.0	Σ=100....				

Rac-**2f**

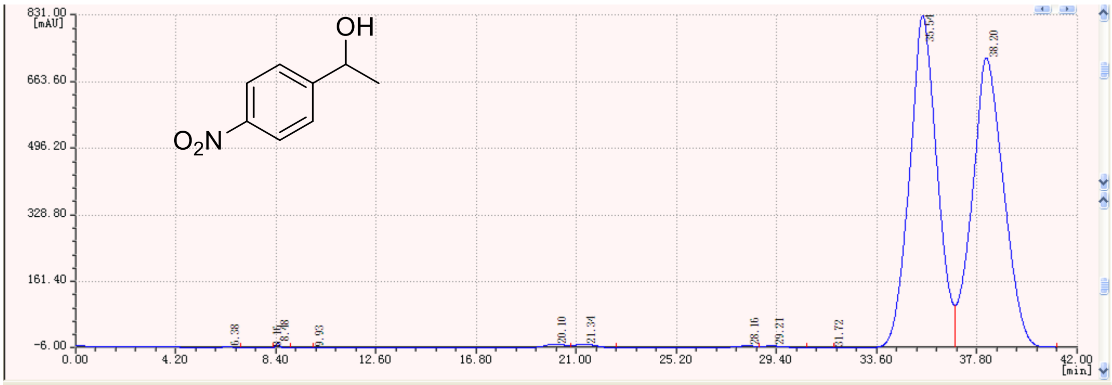

参数设置 分析结果 分组结果 样品信息

ID	组 分 名	RT (分钟)	峰 高	峰 面 积	浓 度	半峰宽 (秒)	分离度	理论塔板数	拖尾因子
1		6.379	82	2257.8	0.0167	25.88	0.00	1210.52	1.01
2		8.162	52	779.6	0.0058	14.09	3.15	6685.10	0.65
3		8.484	1464	12506.1	0.0928	8.03	1.03	22246.61	1.05
4		9.930	7	565.2	0.0042	75.90	1.22	341.20	0.70
5		20.099	910	54043.2	0.4008	55.82	5.45	2583.49	0.79
6		21.337	893	52920.8	0.3925	55.71	0.78	2924.05	1.61
7		28.164	443	26515.8	0.1967	56.26	4.30	4994.01	0.73
8		29.210	427	25307.0	0.1877	55.71	0.66	5479.24	1.94
9		31.719	7	264.9	0.0020	35.57	1.94	15844.99	0.70
10		35.537	83496	6667124.9	49.4505	75.06	2.44	4467.70	0.90
11		38.199	72831	6640124.0	49.2503	85.70	1.17	3959.55	1.34
		总计:	Σ=160612	Σ=134824...	Σ=100....				

(*S*)-**2f**

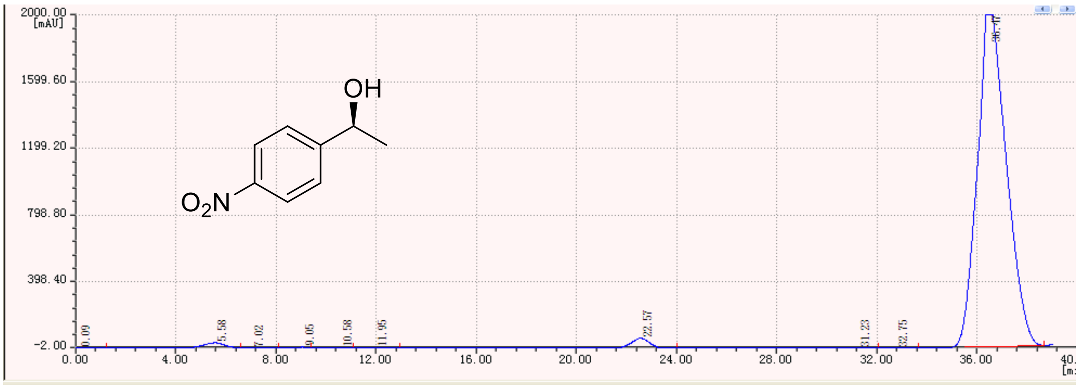

参数设置 分析结果 分组结果 样品信息

ID	组 分 名	RT (分钟)	峰 高	峰 面 积	浓 度	半峰宽 (秒)	分离度	理论塔板数	拖尾因子
1		0.088	19	301.2	0.0018	14.90	0.00	0.70	1.43
2		5.575	3200	197082.5	1.1572	57.89	5.32	184.83	0.85
3		7.018	374	18797.1	0.1104	47.24	0.97	439.83	1.70
4		9.053	863	7407.7	0.0435	8.07	2.60	25092.41	0.77
5		10.582	287	16847.8	0.0989	55.18	1.71	732.93	0.79
6		11.955	478	26474.9	0.1554	52.06	0.90	1050.88	1.07
7		22.565	5991	296427.4	1.7405	46.51	7.60	4691.43	0.95
8		31.227	373	23116.6	0.1357	58.26	5.84	5726.46	0.82
9		32.755	106	5646.1	0.0332	50.07	1.00	8529.72	1.19
10		36.470	214305	16439496.6	96.5235	72.11	2.15	5098.35	1.30
		总计:	Σ=225996	Σ=170315...	Σ=100....				

Rac-**2g**

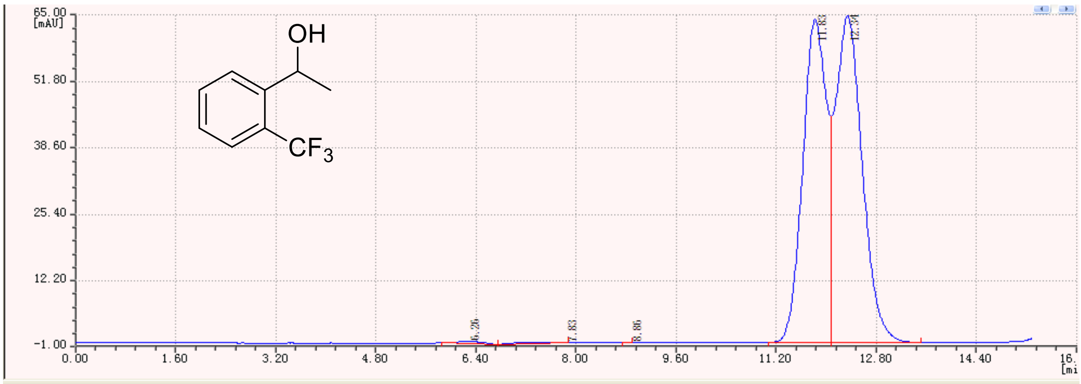

参数设置	分析结果	分組结果	样品信息						
ID	组 分 名	RT (分钟)	峰 高	峰 面 积	浓 度	半峰宽 (秒)	分离度	理论塔板数	拖尾因子
1		6.264	38	1178.4	0.3223	29.15	0.00	920.30	0.93
2		7.834	9	863.6	0.2362	90.19	0.93	150.34	0.53
3		8.864	2	10.5	0.0029	4.94	0.76	64216.46	0.86
4		11.827	6426	171410.2	46.8827	25.07	6.97	4434.37	0.72
5		12.344	6497	192152.7	52.5560	27.80	0.69	3929.13	2.18
		总计:	Σ=12972	Σ=365615.4	Σ=100....				

(*S*)-**2g**

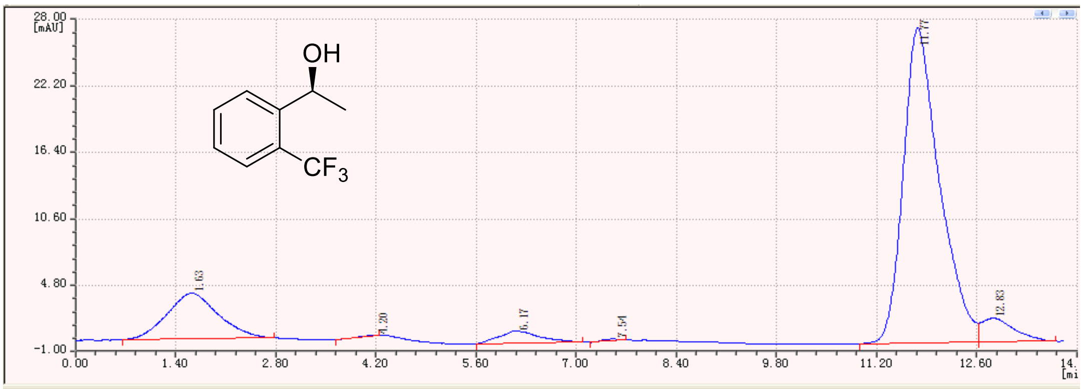

参数设置	分析结果	分組结果	样品信息						
ID	组 分 名	RT (分钟)	峰 高	峰 面 积	浓 度	半峰宽 (秒)	分离度	理论塔板数	拖尾因子
1		1.625	398	19834.8	15.5001	46.85	0.00	23.99	1.18
2		4.204	5	91.1	0.0712	17.13	2.84	1200.43	0.74
3		6.170	107	4189.9	3.2743	36.81	2.57	560.06	1.40
4		7.544	16	256.0	0.2001	15.04	1.87	5013.48	0.86
5		11.774	2751	97082.5	75.8663	33.17	6.19	2510.70	1.21
6		12.834	208	6510.9	5.0880	29.42	1.20	3791.45	2.60
		总计:	Σ=3485	Σ=127965.3	Σ=100....				

Rac-**2h**

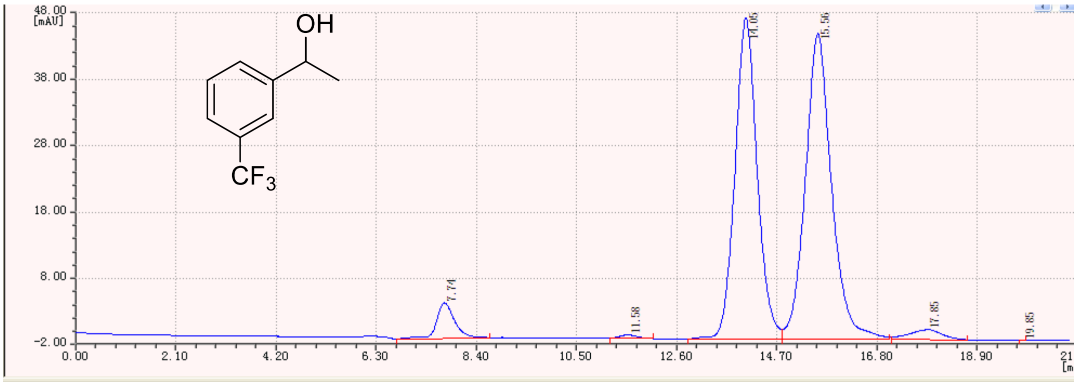

参数设置	分析结果	分组结果	样品信息						
ID	组 分 名	RT(分钟)	峰 高	峰 面 积	浓 度	半峰宽(秒)	分离度	理论塔板数	拖尾因子
1		7.740	533	14002.4	3.9396	24.69	0.00	1957.87	1.05
2		11.585	51	1251.8	0.3522	23.07	5.68	5024.55	1.03
3		14.055	4835	156777.4	44.1098	30.48	3.26	4237.93	0.90
4		15.565	4606	176157.0	49.5623	35.95	1.60	3736.00	1.51
5		17.853	150	7225.3	2.0329	45.28	1.99	3098.61	1.04
6		19.847	3	11.5	0.0032	3.60	2.88	607102.92	1.06
		总计:	Σ=10178	Σ=355425.4	Σ=100....				

(*S*)-**2h**

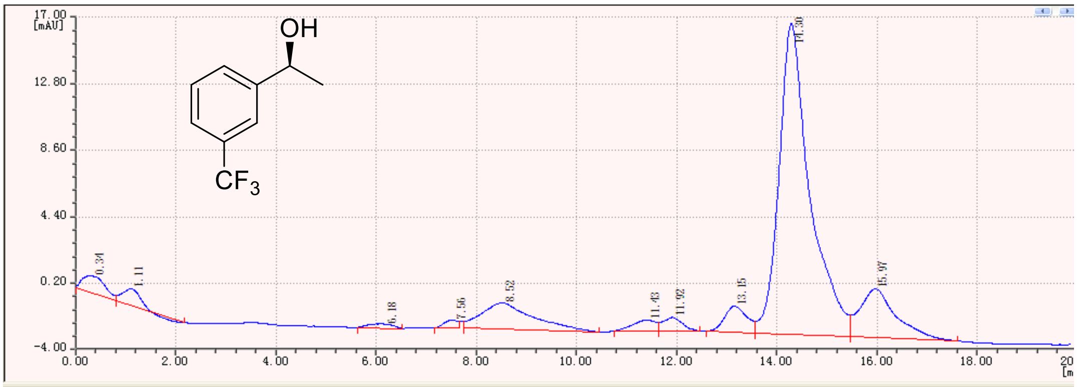

参数设置	分析结果	分组结果	样品信息						
ID	组 分 名	RT(分钟)	峰 高	峰 面 积	浓 度	半峰宽(秒)	分离度	理论塔板数	拖尾因子
1		0.342	97	3409.2	2.6424	33.04	0.00	2.13	1.02
2		1.107	104	2610.4	2.0233	23.59	0.95	43.88	0.97
3		6.184	25	857.8	0.6648	32.25	6.42	732.67	0.76
4		7.555	48	923.7	0.7160	18.09	1.92	3476.68	0.64
5		8.524	166	12049.9	9.3397	68.23	0.79	311.01	1.52
6		11.427	66	2171.4	1.6830	30.93	2.07	2721.11	0.66
7		11.922	84	2239.7	1.7359	25.06	0.62	4509.87	1.45
8		13.152	162	5119.6	3.9682	29.71	1.59	3906.71	0.88
9		14.297	1962	84058.2	65.1524	40.27	1.16	2511.91	1.31
10		15.972	308	15577.9	12.0742	47.54	1.35	2249.47	1.69
		总计:	Σ=3022	Σ=129017.7	Σ=100....				

Rac-**2i**

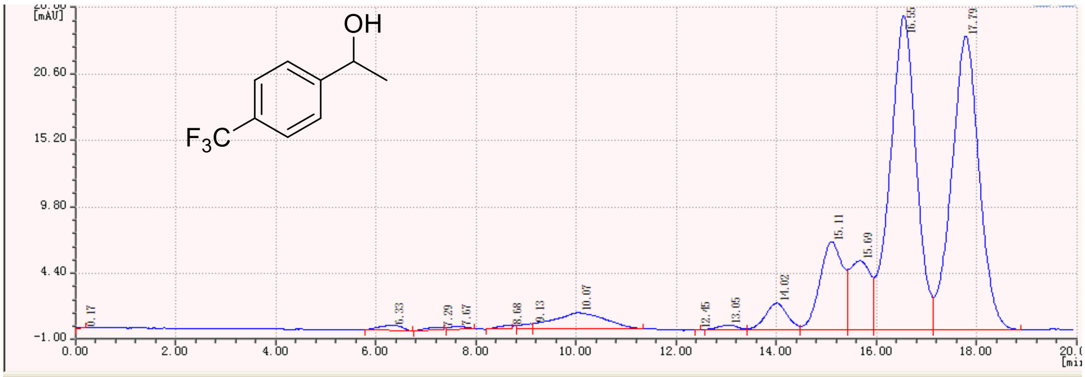

参数设置 分析结果 分组结果 样品信息

ID	组 分 名	RT(分钟)	峰 高	峰 面 积	浓 度	半峰宽(秒)	分离度	理论塔板数	拖尾因子
2		6.332	40	1294.3	0.5331	30.42	11.65	863.59	0.80
3		7.293	19	527.3	0.2172	26.09	1.20	1557.53	0.58
4		7.670	26	604.2	0.2489	21.84	0.55	2457.18	1.06
5		8.675	27	537.7	0.2215	18.72	1.75	4280.36	0.56
6		9.130	43	715.1	0.2946	15.63	0.93	6797.53	0.52
7		10.070	130	9494.2	3.9106	68.65	0.79	428.82	1.18
8		12.447	3	17.6	0.0072	5.51	2.26	101795.89	0.85
9		13.055	41	1166.5	0.4805	26.74	1.33	4748.91	0.87
10		14.018	220	6771.7	2.7892	28.93	1.22	4678.48	0.88
11		15.111	717	22851.5	9.4124	29.96	1.31	5070.97	0.74
12		15.686	561	16341.8	6.7311	27.38	0.71	6541.04	1.00
13		16.547	2552	91737.4	37.7861	33.79	0.99	4779.21	1.00
14		17.790	2385	90699.2	37.3585	35.75	1.26	4936.09	1.20
		总计:	Σ=6767	Σ=242780.8	Σ=100....				

(*S*)-**2i**

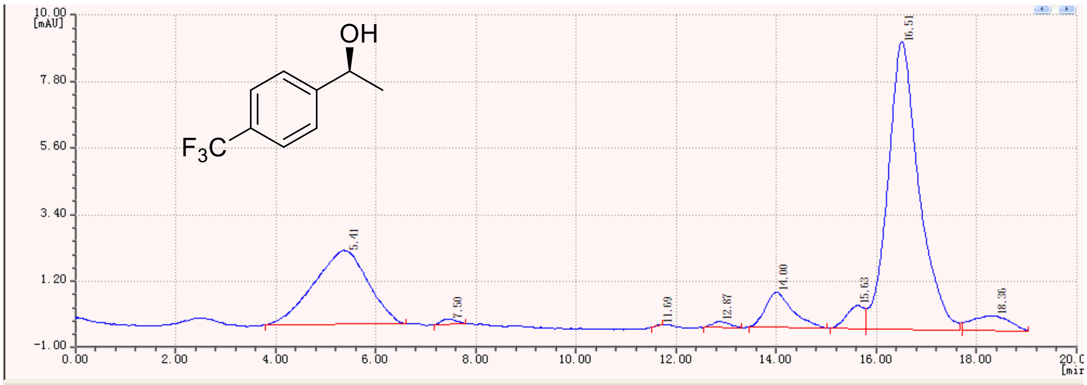

参数设置 分析结果 分组结果 样品信息

ID	组 分 名	RT(分钟)	峰 高	峰 面 积	浓 度	半峰宽(秒)	分离度	理论塔板数	拖尾因子
1		5.412	245	18474.6	26.7592	70.88	0.00	116.18	0.89
2		7.502	18	373.6	0.5411	19.51	1.63	2946.57	1.03
3		11.687	3	21.8	0.0316	6.83	11.21	58320.97	0.83
4		12.867	20	464.3	0.6725	21.82	2.91	6928.21	1.18
5		14.003	115	4339.5	6.2854	35.47	1.40	3106.42	1.34
6		15.630	76	1865.6	2.7022	23.07	1.96	9144.47	0.65
7		16.512	950	40864.1	59.1888	40.43	0.98	3323.67	1.31
8		18.362	49	2636.7	3.8190	50.58	1.43	2626.51	1.01
		总计:	Σ=1476	Σ=69040.2	Σ=100....				

Rac-**2m**

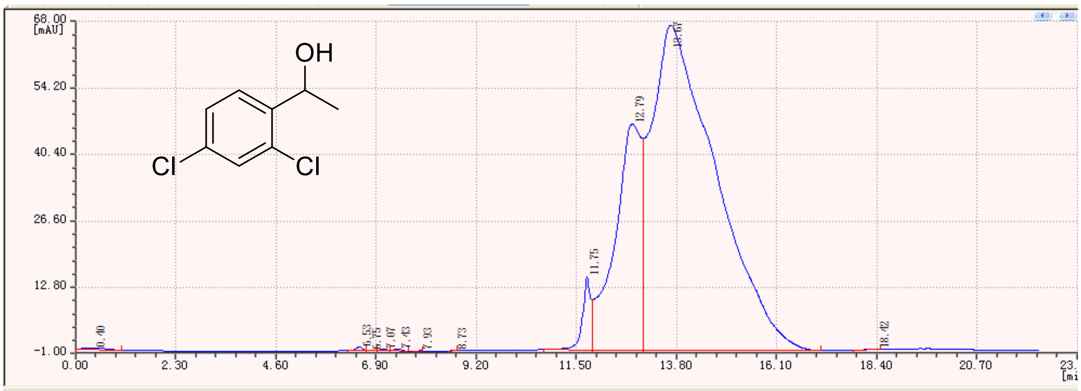

参数设置 分析结果 分组结果 样品信息

ID	组 分 名	RT (分钟)	峰 高	峰 面 积	浓 度	半峰宽 (秒)	分离度	理论塔板数	拖尾因子
1		0.398	41	1421.6	0.1555	32.59	0.00	2.97	1.10
2		6.525	81	805.8	0.0881	9.35	10.31	9702.93	0.76
3		6.750	17	188.3	0.0206	10.41	0.80	8379.29	1.60
4		7.072	35	362.2	0.0396	9.73	1.13	10532.52	0.99
5		7.427	42	526.6	0.0576	11.79	1.16	7914.46	0.81
6		7.927	5	53.1	0.0058	9.97	1.62	12585.61	0.77
7		8.727	3	16.6	0.0018	5.21	3.72	55875.70	0.88
8		11.750	1523	19472.0	2.1294	12.02	12.39	19051.33	0.56
9		12.793	4685	198189.7	21.6736	39.76	1.42	2062.94	0.63
10		13.672	6745	693223.5	75.8095	96.61	0.45	399.14	2.92
11		18.418	5	168.6	0.0184	31.71	2.61	6725.78	0.64
		总计:	Σ=13182	Σ=914428.1	Σ=100....				

(*S*)-**2m**

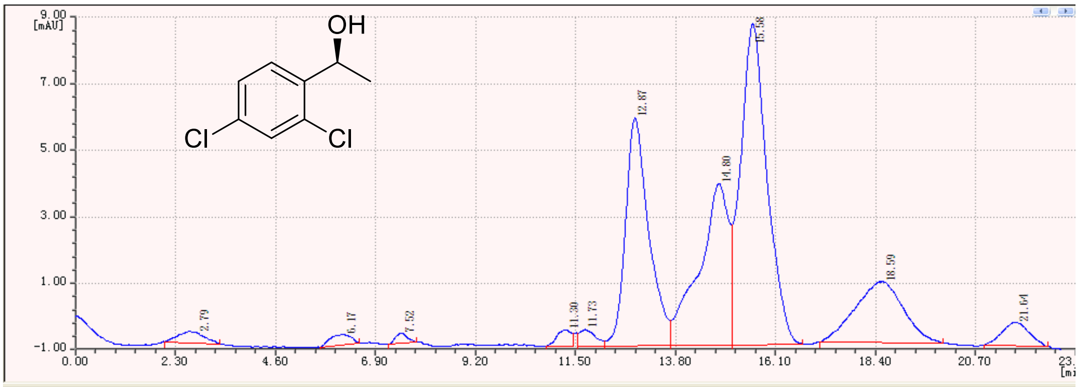

参数设置 分析结果 分组结果 样品信息

ID	组 分 名	RT (分钟)	峰 高	峰 面 积	浓 度	半峰宽 (秒)	分离度	理论塔板数	拖尾因子
1		2.792	33	1391.9	1.2472	39.65	0.00	98.81	0.81
2		6.170	30	1014.8	0.9094	31.80	3.34	750.41	0.90
3		7.520	29	582.1	0.5216	18.87	1.88	3166.47	1.12
4		11.300	48	1154.9	1.0349	22.62	6.43	4975.21	0.67
5		11.727	49	1233.3	1.1052	23.66	0.65	4895.96	1.82
6		12.865	683	25487.5	22.8388	35.08	1.37	2680.91	1.10
7		14.798	485	22894.6	20.5154	44.37	1.72	2216.76	0.64
8		15.580	965	40701.7	36.4720	39.65	0.66	3077.73	1.65
9		18.587	182	14094.4	12.6297	72.80	1.89	1299.35	0.94
10		21.637	71	3042.0	2.7259	40.27	1.90	5752.40	0.94

Rac-**2n**

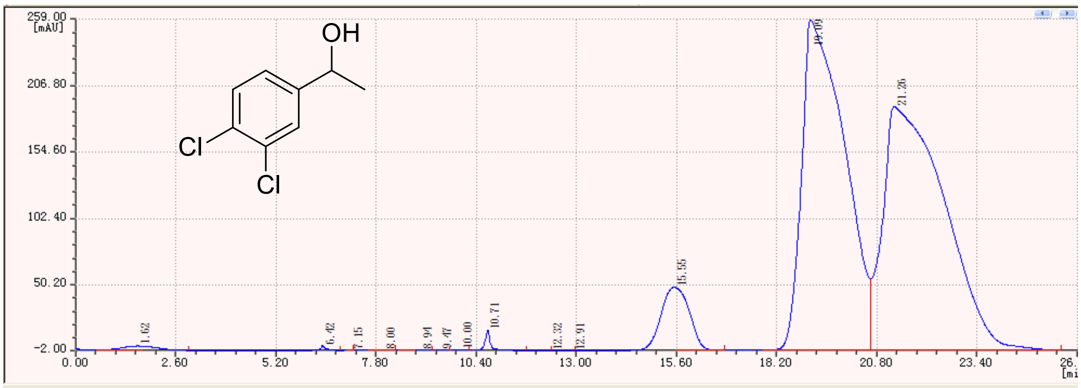

参数设置	分析结果	分組结果	样品信息

ID	組 分 名	RT (分钟)	峰 高	峰 面 积	浓 度	半峰宽 (秒)	分离度	理论塔板数	拖尾因子
1		1.615	329	20492.3	0.4597	58.55	0.00	15.17	1.01
2		6.417	371	3700.8	0.0830	9.38	4.99	9334.38	1.04
3		7.147	7	145.7	0.0033	19.56	1.78	2660.56	0.76
4		7.995	59	1480.4	0.0332	23.59	1.39	2290.18	0.90
5		8.939	64	2397.2	0.0538	35.21	1.13	1284.63	0.78
6		9.472	45	843.1	0.0189	17.61	0.71	5765.55	1.44
7		10.004	56	1159.9	0.0260	19.47	1.01	5261.40	0.87
8		10.707	1592	16491.7	0.3700	9.74	1.70	24097.04	1.47
9		12.317	2	21.0	0.0005	9.87	5.80	31047.49	0.78
10		12.907	3	50.2	0.0011	15.72	1.63	13431.60	0.91
11		15.549	4942	251302.7	5.6374	47.80	2.94	2108.96	1.08
12		19.092	25982	2010842.7	45.1088	72.75	2.07	1372.64	1.47
13		21.262	19129	2148832.3	48.2043	105.59	0.86	808.08	3.53
		总计:	Σ=52581	Σ=4457760.0	Σ=100....				

(*S*)-**2n**

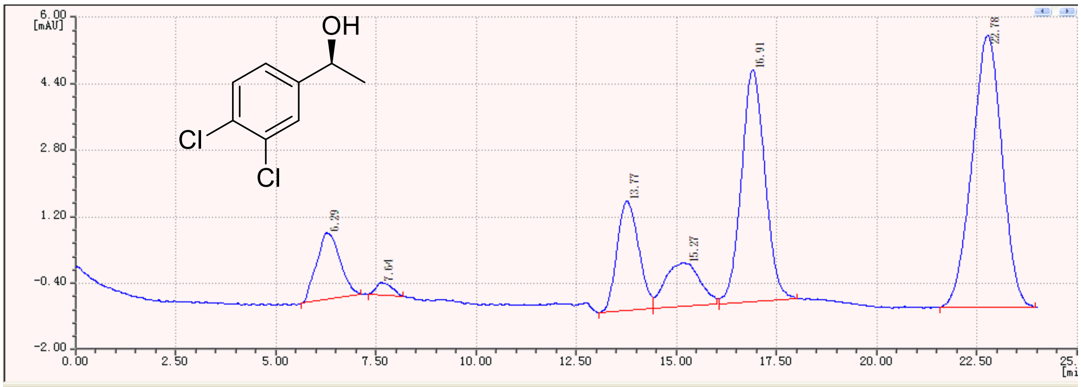

参数设置	分析结果	分組结果	样品信息

ID	組 分 名	RT (分钟)	峰 高	峰 面 积	浓 度	半峰宽 (秒)	分离度	理论塔板数	拖尾因子
1		6.287	160	6494.3	8.0512	38.15	0.00	541.17	1.28
2		7.639	30	814.5	1.0098	25.52	1.50	1785.65	1.18
3		13.765	263	9890.8	12.2620	35.35	7.10	3021.99	1.07
4		15.265	105	6341.9	7.8623	56.78	1.15	1440.86	0.94
5		16.910	558	22965.7	28.4716	38.69	1.22	3807.90	1.16
6		22.775	653	34154.7	42.3430	49.17	4.71	4276.82	1.00
		总计:	Σ=1769	Σ=80661.9	Σ=100....				
